# Nuclear Receptor Nur77 Deficiency Alters Dendritic Cell Function

**DOI:** 10.3389/fimmu.2018.01797

**Published:** 2018-08-03

**Authors:** Nina Tel-Karthaus, Esther D. Kers-Rebel, Maaike W. Looman, Hiroshi Ichinose, Carlie J. de Vries, Marleen Ansems

**Affiliations:** ^1^Department of Radiation Oncology, Radiotherapy & OncoImmunology Laboratory, Radboud Institute for Molecular Life Sciences, Radboud University Medical Center, Nijmegen, Netherlands; ^2^School of Life Science and Technology, Tokyo Institute of Technology, Yokohama, Japan; ^3^Department of Medical Biochemistry, Academic Medical Center, Amsterdam Cardiovascular Sciences, Amsterdam, Netherlands

**Keywords:** dendritic cells, dendritic cell-based immunotherapy, nuclear receptors, NR4A, Nur77

## Abstract

Dendritic cells (DCs) are the professional antigen-presenting cells of the immune system. Proper function of DCs is crucial to elicit an effective immune response against pathogens and to induce antitumor immunity. Different members of the nuclear receptor (NR) family of transcription factors have been reported to affect proper function of immune cells. Nur77 is a member of the NR4A subfamily of orphan NRs that is expressed and has a function within the immune system. We now show that Nur77 is expressed in different murine DCs subsets *in vitro* and *ex vivo*, in human monocyte-derived DCs (moDCs) and in freshly isolated human BDCA1^+^ DCs, but its expression is dispensable for DC development in the spleen and lymph nodes. We show, by siRNA-mediated knockdown of Nur77 in human moDCs and by using Nur77^−/−^ murine DCs, that Nur77-deficient DCs have enhanced inflammatory responses leading to increased T cell proliferation. Treatment of human moDCs with 6-mercaptopurine, an activator of Nur77, leads to diminished DC activation resulting in an impaired capacity to induce IFNγ production by allogeneic T cells. Altogether, our data show a yet unexplored role for Nur77 in modifying the activation status of murine and human DCs. Ultimately, targeting Nur77 may prove to be efficacious in boosting or diminishing the activation status of DCs and may lead to the development of improved DC-based immunotherapies in, respectively, cancer treatment or treatment of autoimmune diseases.

## Introduction

Dendritic cells (DCs) are professional antigen-presenting cells. An important function of DCs is to instruct T cells to elicit immunity or tolerance (1, 2). Many factors contribute to the way DCs are shaped to elicit this function. Important factors are the type of pathogens that DCs encounter, such as bacteria or viruses, but also different microenvironmental factors in the tissues they reside in play a crucial role. DCs can be subdivided into classical or conventional DCs (cDC), interferon-producing plasmacytoid DCs (pDC), and monocyte-derived DCs (moDC) each with their own specialized function (3–5). Because of their crucial role in the immune system, different subsets of DCs are exploited in immune therapy (6–15). So far, treatment success is limited and functional knowledge on how DCs initiate and stably steer antitumor responses *in vivo* is important (13–15). Identification of transcription factors that control DC function in both immunity and tolerance is highly relevant, as these factors may serve as targets to modulate DC activity and function for the development of more successful DC-based immunotherapies.

Different members of the nuclear receptor (NR) family of transcription factors and their ligands have been shown to affect immune cells, including DCs (16–20). NRs are ligand inducible transcription factors having among others, steroid hormones or cellular metabolites as ligands. Several members have been well studied and were shown to play an immune modulatory role in DCs. Another group of NRs are so called “orphan” NRs for which no natural ligand has been identified yet, and the existence of ligands is disputed. The NR4A subfamily of orphan receptors comprises three members, namely, Nur77 (NR4A1/TR3/NGFI-B), Nurr1 (NR4A2/NOT/TINUR), and NOR-1 (NR4A3/TEC/MINOR). Their activity appears to be primarily regulated at the expression level. The expression of the NR4As can be induced by a diverse range of signals, including fatty acids, stress, growth factors, cytokines, peptide hormones, and physical stimuli (21). Hallmark of this subfamily is to respond quickly to such changes in cellular environments and regulate gene expression in a ligand-independent manner.

Members of this subfamily have been shown to be involved in a wide variety of pathological conditions. They have been shown to be dysregulated in multiple cancer types and promote or suppress tumors depending on specific cellular and tissue context, subcellular localization, external stimuli, protein–protein interactions, and post-translational modifications in cancer cells [reviewed in Ref. (22)]. In addition, there is also increasing evidence that the NR4As play a role in neurodegenerative disorders such as Alzheimer’s and Parkinson’s disease by contributing to neuronal cell death *via* modulating mitochondrial function and ER stress by controlling intracellular levels of ROS and Ca^2+^ and regulating cellular autophagy (23–26). Also in autoimmune-driven central nervous system (CNS) inflammation, the NR4A NRs have been shown to play an important role (27, 28).

NR4A receptors have emerged to play an important role within the immune balance by transcriptional regulation of cytokines and growth factors in macrophages (29, 30). In addition, they have been shown to be involved in the negative selection of self-reactive T cell clones in the thymus (31, 32) and are essential for thymic regulatory T cell development (33). Studies in Nur77^−/−^ mice imply that Nur77 functions as a master regulator in the differentiation and survival of Ly-6C^−^ monocytes (34, 35). Ly-6C^+^ and Ly-6C^−^ monocytes that do express Nur77 do not develop into moDCs (36). Thus, Nur77 expression is not required for the development into moDCs but is for differentiation of Ly-6C^+^ monocytes into Ly-6C^−^ “patrolling” monocytes (34, 36). Moreover, Nur77 has been shown to be involved in the polarization of macrophages toward an inflammatory phenotype important in atherosclerosis (37, 38).

We and others have recently reported expression of Nur77, Nurr1, and NOR-1 in murine DCs (39–43). Nurr1 has been shown to restrict the immunogenicity of bone marrow derived DCs (BMDCs) (43) and NOR-1 leads to activation-induced cell death in DCs (39), is important in DC migration (42), and is involved in TLR-mediated activation and gene expression of DCs (44). However, so far, the role of Nur77 expression in DCs remains elusive. We here set out to assess the expression kinetics and function of Nur77 in multiple subsets of murine and human DCs and its subsequent effect on inducing T cell activation, revealing a function as activation modulator for Nur77 in DCs. Knowledge regarding the possibilities in altering the activation status of DCs may prove to be beneficial in improving DC-based vaccination strategies.

## Materials and Methods

### Mice

6- to 16-week-old C57BL/6J and Balb/C mice (Charles River), Nur77^−/−^ mice (45) on a C57BL/6 background, and Nur77^GFP^ mice [016607; C57BL/6-Tg(Nr4a1-EGFP/cre)820Khog/J; Jackson Laboratory] were housed under specific pathogen-free conditions in individually ventilated cage units at the Central Animal Laboratory (Nijmegen, The Netherlands). Standard laboratory chow and sterile drinking water were provided *ad libitum*. All animal experiments were approved by the Radboud University’s Animal Welfare Body (*Instantie voor Dierenwelzijn IvD*) and the Animal Experiment Committee (*DierExperimentenCommissie, RUDEC*) that is recognized by the CCD (Central Authority for Scientific Procedures on Animals). The experiments were performed according to institutional, national, and European guidelines as stipulated in the *Wet op de dierproeven* and in the *Dierproevenbesluit*.

### *In Vitro* Generation of Murine DCs

DCs were generated from murine BM isolated from the femur/tibia of the mice. To obtain pDCs and cDCs, cells were cultured for 8–10 days (37°C, 10% CO_2_) in RPMI 1640 supplemented with 10% fetal calf serum (Gibco-BRL Life Technologies), 0.5% antibiotic–antimycotic (Gibco/Invitrogen), 1% ultra-glutamine (Lonza), 50 µM β-mercaptoethanol (Sigma-Aldrich), and 200 ng/ml human rFlt3L (PeproTech). Pure cell populations were isolated by labeling single cell suspensions with anti-SiglecH-FITC (eBiosciences) and anti-CD11c-APC antibodies for pDCs and cDCs, respectively. pDCs were positively sorted with anti-FITC microbeads, the negative fraction was subjected to positive selection with anti-APC microbeads (both Miltenyi Biotec, Germany) to obtain cDCs as described previously (40). CD103^+^ murine DCs were generated by culturing BM cells in RPMI 1640 supplemented with 10% FCS, 0.5% antibiotic–antimycotic, 1% ultra-glutamine, 50 µM β-mercaptoethanol, 5 ng/ml mGM-CSF, and 200 ng/ml human rFlt3L, fresh medium was added at day 6, and cells were replated in fresh medium at day 9. Cells were harvested and used for experiments at day 14. The purity of the isolated DC subsets was ensured by flow cytometry.

### Tumor Induction

The transgenic cell line 9464D was derived from spontaneous tumors from TH-MYCN transgenic mice on C57BL/6 background and were a kind gift from Dr. Orentas (NIH, Bethesda, MD, USA). 9464D cells were cultured in DMEM containing 10% fetal calf serum, 1% non-essential amino acids, 0.5% antibiotic–antimycotic, and 50 µM β-mercaptoethanol. For induction of tumors, 1 × 10e6 9464D cells were injected s.c. in 100 µl PBS on the right flank of the mice. Tumor growth was measured every 3–4 days using calipers. Spleen and lymph nodes (LNs) of the mice were taken when the tumor was more than 5 mm in diameter.

### Flow Cytometry

To obtain single cells for flow cytometric staining, murine spleen was passaged over a 100 µm cell strainer, and murine LNs were incubated in serum-free medium containing collagenase (Worthington) and DNAseI (Roche), later supplemented with 1 mM EDTA. *In vitro* generated human and murine DCs, and *ex vivo* isolated murine spleen and LN cells were stained using standard antibody staining protocols with antibodies listed in Table S1 in Supplementary Material. Cell viability was assessed by staining with fixable viability dye eFluor™ 450 (eBioscience). Samples were acquired on a FACS Verse (BD Bioscience), and data were analyzed with FlowJo software (Tree Star).

### ELISA

Human and mouse IL-6, TNFα, IL-12p70, and human IFNγ present in the supernatant of DC cultures was measured using the ELISA kit (Thermo Fisher) according to the manufacturers protocol.

### Murine Type I IFN Bioassay

Type I IFN activity in the supernatant of murine pDCs was measured using L929 cells transfected with an interferon-sensitive luciferase construct (ISRE-L929) (46) with reference to a recombinant mouse IFN-β standard (Sigma). In short, pDC culture supernatants were added to ISRE-L929 IFN reporter cells and incubated for 4–6 h. Then, the cells were lysed in Passive Lysis Buffer (Promega), mixed with firefly luciferin substrate (Promega), and measured on a Victor3 Luminometer.

### Mixed Leukocyte Reaction (MLR) Murine DCs

After 16–24 h of stimulation with 1 µg/ml CpGB (1668, Sigma-Aldrich) or 4 µg/ml R848, pDCs, cDCs, or CD103^+^ DCs were washed and co-incubated with carboxyfluorescein diacetate succinimidyl ester (CFSE)-labeled allogeneic BalB/C T cells. T cells were isolated using the T cell isolation kit (EasySep). The cells were co-incubated for 3 days in round-bottom 96-well cluster plates (Corning). T cell proliferation was measured by CFSE dilution by FACS.

### Generation of Human DCs

DCs were generated from cells isolated from buffy coats obtained from healthy volunteers (Sanquin, Nijmegen, The Netherlands) after written informed consent as per the Declaration of Helsinki. The study was approved by the Institutional Review Board of the Radboud University Nijmegen Medical Center, Commissie Mensgebonden Onderzoek. Peripheral blood mononuclear cells (PBMCs) were purified *via* Ficoll density gradient centrifugation (Lucron Bioproducts). moDCs were cultured as described previously (47). In short, plastic-adherent monocytes were cultured for 6 days in RPMI 1640 medium with 1% ultra-glutamine, 0.5% antibiotic–antimycotic, 10% (v/v) fetal calf serum, 300 U/ml IL4, and 450 U/ml GM-CSF (both Cellgenix). IL4 and GM-CSF were added again at day 3. To obtain fresh human myeloid dendritic cells (mDCs), CD14^+^ cells were depleted from the PBMCs followed by BDCA1^+^ DC isolation using the CD1c (BDCA1)^+^ Dendritic Cell Isolation Kit (Miltenyi Biotec). Purity of the freshly isolated mDCs was ensured by flow cytometry.

### Small Interfering RNA-Mediated Knockdown

For Nur77 silencing in human moDCs, the ON-TARGETplus SMARTpool NR4A1 (Dharmacon) containing four different Nur77 targeting siRNA oligos each 19 nt long was used. The irrelevant siRNA ON-TARGETplus Non-Targeting siRNA#1 (Dharmacon) was used as control. moDCs were electroporated at day 4 as described before (47). Electroporated DCs were stimulated with 1 µg/ml LPS (Sigma) or 4 µg/ml R848 (Enzo Life Sciences) at day 6. Supernatant was taken 24 h later.

### RNA Isolation and Quantitative PCR

Total RNA was isolated and cDNA was synthesized as described before (47). mRNA levels for the genes of interest were determined with a CFX96 sequence detection system (Bio-Rad) using the Faststart SYBR green mastermix (Roche) with SYBR Green as the fluorophore and gene-specific oligonucleotide primers. The primers for human porphobilinogen deaminase (PBGD), IL-6, TNFα, and IL-12 (47) and murine PBGD, TLR7, and TLR9 (40) were described previously. Other primers used (forward and reverse) are listed in Table S2 in Supplementary Material. Reaction mixtures and program conditions were used that were recommended by the manufacturer (Bio-Rad). Quantitative PCR data were analyzed with the CFX Manager V1.6.541.1028 software (Bio-Rad) and checked for correct amplification and dissociation of the products. As we described previously for human and murine DCs, mRNA levels of the genes of interest were normalized to mRNA levels of the housekeeping gene PBGD (19, 20, 40, 47, 48) and were calculated according to the cycle threshold method (49).

### Human MLR

Human day 6 moDCs were pretreated with 1 or 10 µM 6-mercaptopurine (6-MP) (Sigma) or vehicle control (DMSO) for 8 h, before o/n stimulation with 4 µg/ml R848. At day 7, the medium was replaced with fresh DC medium, and allogeneic peripheral blood lymphocytes were added to the DCs, in a ratio of 1:10 (DCs:T cells) and cocultured for 144 h. Supernatant was taken for IFNγ measurements.

### Statistical Analysis

In each experiment, at least three mice or human donors were used to be able to perform statistical testing. Each legend contains the information of the number of mice or human donors used including the statistics that was used to calculate significance. Statistical testing was performed using GraphPad Prism 5 software (GraphPad, La Jolla, CA, USA). A *P* < 0.05 was considered significant.

## Results

### Nur77 Expression and Function in Murine DCs

**Figure 1:**
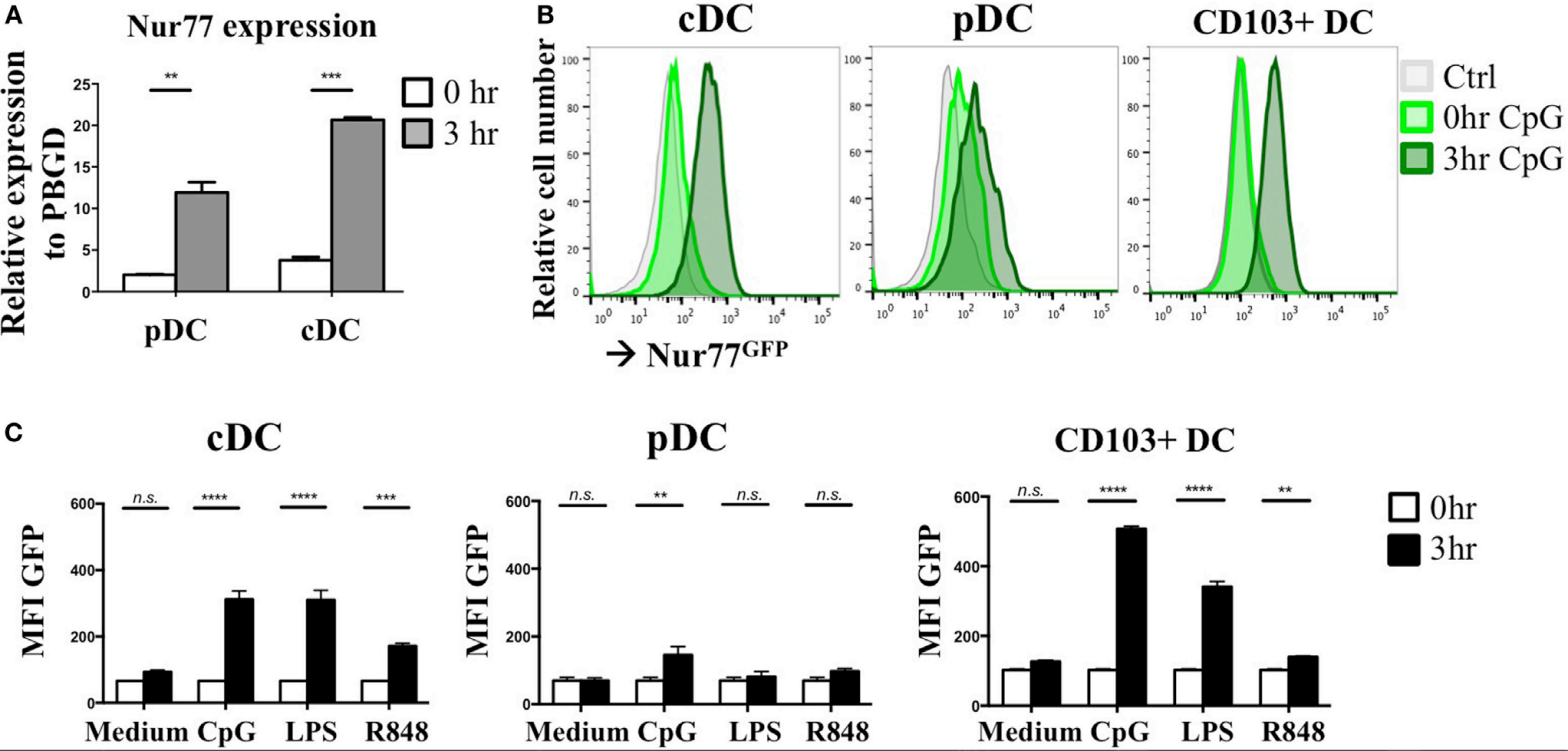
Nur77 expression level in *in vitro* generated murine dendritic cells (DCs). **(A)** Plasmacytoid DCs (pDCs) and conventional DCs (cDCs) were sorted from FLT3L bone marrow cultures pooled from three mice per experiment and immediately lysed for RNA isolation (0 h) or stimulated for 3 h with a combination of R848 and CpG. mRNA expression levels of Nur77 were detected by qPCR analysis. Data shown are the mean of three independent experiments ± SEM, two-tailed unpaired *t*-test: *P < 0.05; **P < 0.01; and ***P < 0.001. **(B)** *In vitro* generated cDCs, pDCs, or CD103^+^ DCs from Nur77^GFP^ or control mice were stimulated for the indicated times with CpG, and GFP expression was determined by flow cytometry. Shown are the representative data of three mice. **(C)** Quantification of Nur77^GFP^ expression in cDC, pDC, or CD103^+^ DC stimulated with CpG, LPS, or R848 for 0 or 3 h, presented as the geometric mean (MFI) ± SEM, two-way ANOVA with Sidak’s multiple comparisons test (*n* = 3). *n.s*., not significant; *P < 0.05; **P < 0.01; ***P < 0.001; and ****P < 0.0001.

As NR4A NRs are typical early response genes induced upon stimulation (29), we tested Nur77 expression in cDCs and pDCs 3 h after stimulation with a combination of the TLR7/8 ligand R848 and the TLR9 ligand CpG. DCs were differentiated from murine BM *in vitro* with FLT3L as this reflects physiologic DC development and gives rise to a mixture of both cDCs and pDCs (50). pDCs were detected as CD11c^pos^B220^pos^SiglecH^pos^ and cDCs were defined as CD11c^pos^B220^neg^SiglecH^neg^ and were sorted and stimulated as described before (40) (Figure S1A in Supplementary Material). In agreement with its classification as early response gene, Nur77 mRNA levels were strongly upregulated after 3 h of stimulation in murine pDCs as well as cDCs compared with freshly sorted cells (0 h) (Figure 1A). To further assess Nur77 expression kinetics, we used BM cells from transgenic Nur77 reporter mice, where the induction of the Nur77 promoter drives GFP expression (Nur77^GFP^) (51). In line with its mRNA expression, cDCs and to a lesser extend pDCs up regulate Nur77^GFP^ already after 3 h of stimulation with CpG (Figure 1B). In addition to FLT3L-derived BMDCs, we tested Nur77^GFP^ in BMDCs differentiated into Batf3-dependent CD103^+^ DCs (CD11c^pos^B220^neg^CD103^pos^) when cultured with GM-CSF and FLT3L (52) (for gating strategy see Figure S1B in Supplementary Material). In CD103^+^ DCs, there is also already prominent expression of Nur77^GFP^ after 3 h stimulation with CpG (Figure 1B). Our data further show that the expression in cDCs was highest after CpG and LPS stimulation, whereas the expression was less pronounced in response to R848. pDC and CD103^+^ DCs revealed highest expression of Nur77^GFP^ after stimulation with CpG, compared with LPS and R848 (Figure 1C). These data indicate that in different types of *in vitro* generated DCs, Nur77 expression is quickly induced upon stimulation with inflammatory ligands, and that the expression in response to TLR-specific agonists varies in different DC subsets.

### Nur77 Does Not Have a Major Impact on the Development of Murine DCs in Spleen and LNs

**Figure 2:**
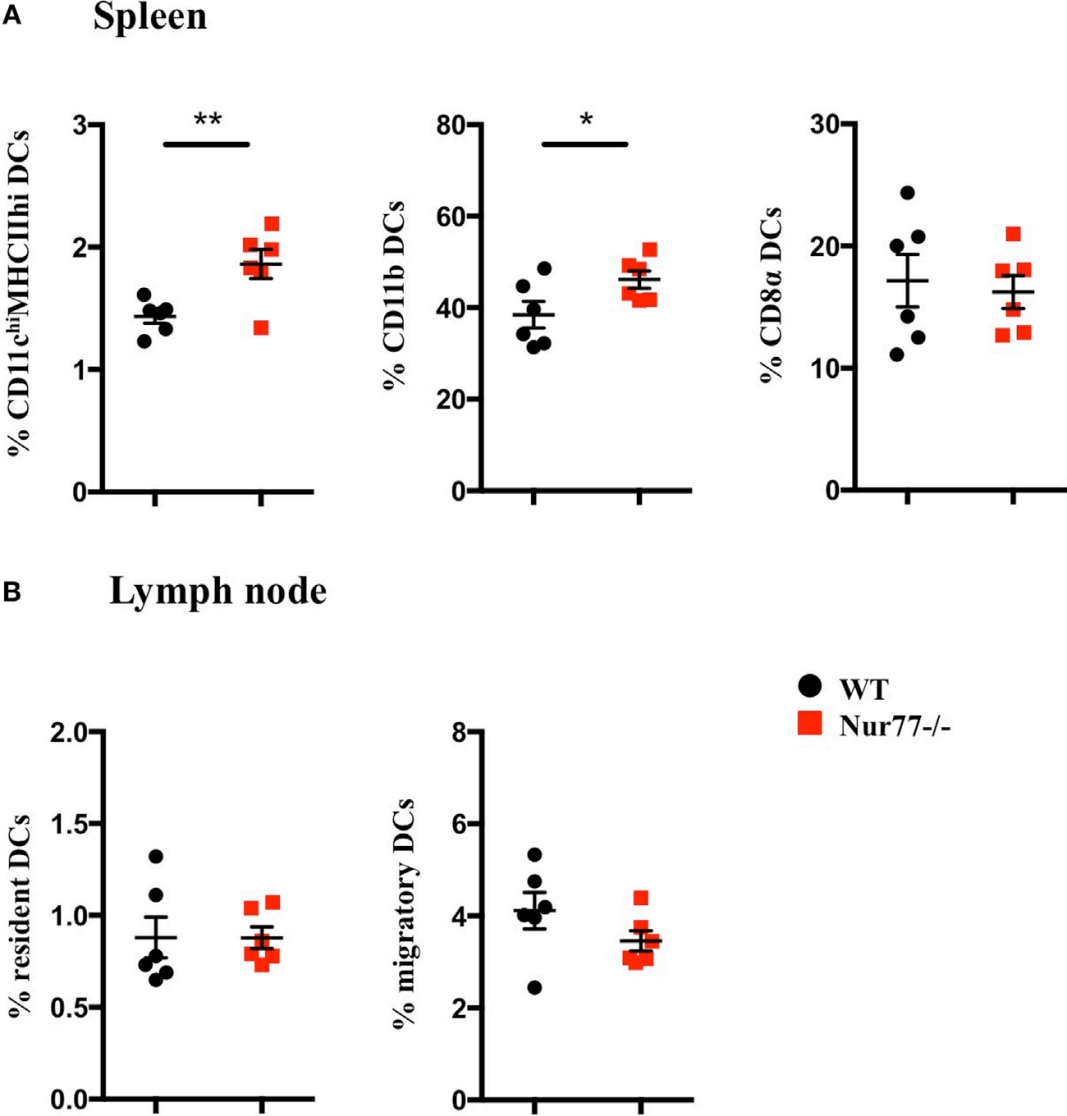
Absence of Nur77 expression does not have a major impact on dendritic cell (DC) development in the spleen and lymph nodes (LNs). % of DCs in **(A)** spleen and **(B)** inguinal LNs of WT or Nur77^−/−^ mice. Shown are the means of pooled data of two independent experiments ± SEM for WT or Nur77^−/−^ mice (*n* = 3). Two-tailed unpaired *t*-test: *P < 0.05; **P < 0.01; and ***P < 0.001.

**Figure 3:**
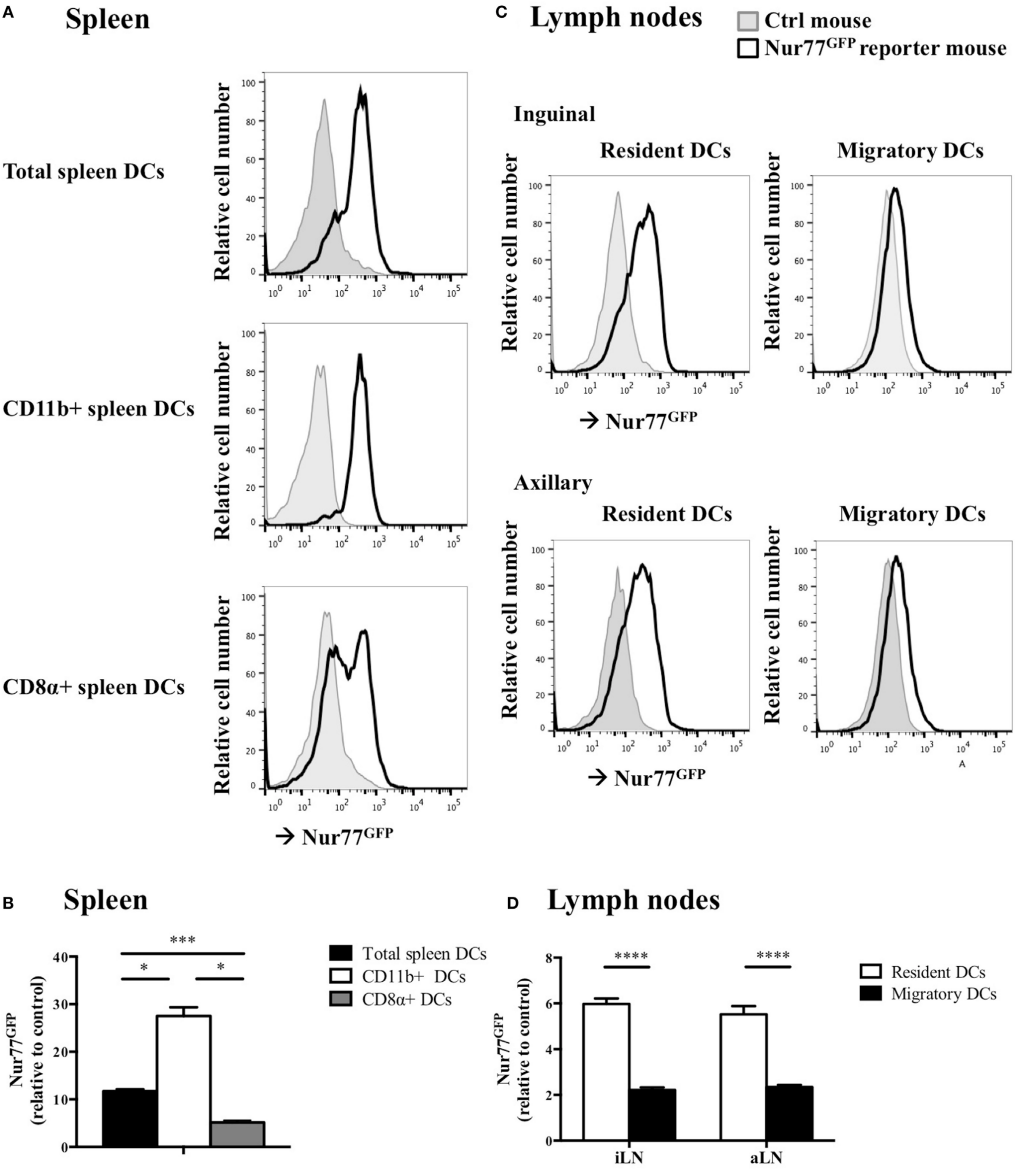
Nur77 expression level in murine dendritic cells (DCs) *ex vivo*. **(A)** *Ex vivo* Nur77^GFP^ expression in the different DC subsets in spleen, shown are the representative data of one out of three transgenic Nur77 reporter mice, where the induction of the Nur77 promoter drives GFP expression (Nur77^GFP^) mice. **(B)** Quantification of Nur77^GFP^ expression in the different DC subsets in the spleen, presented as the relative Nur77^GFP^ expression to control mice ± SEM, one-way ANOVA with Sidak’s multiple comparisons test (*n* = 3). **(C)** *Ex vivo* Nur77^GFP^ expression in the resident and migratory DC subsets of the inguinal and axillary lymph nodes (aLN), shown are the representative data of one out of three transgenic Nur77 reporter mice. **(D)** Quantification of Nur77^GFP^ expression in the resident and migratory DC subsets of the inguinal (iLN) and aLN, presented as the relative Nur77^GFP^ expression to control mice ± SEM, two-way ANOVA with Sidak’s multiple comparisons test (*n* = 3): *P < 0.05; ***P < 0.001; and ****P < 0.0001.

To test whether Nur77 expression is required for the development of DCs, we investigated the presence of different DC subsets in spleen and LNs of WT and Nur77^−/−^ mice. We observed a small but significant increase in the percentage of total CD11c^hi^MHCII^hi^ DCs and in CD11b^+^ DCs (CD11c^hi^MHCII^hi^Sirpα^pos^CD24^neg^CD115^neg^CD4^pos^) of the spleen of Nur77^−/−^ mice relative to WT mice (Figure 2A; Figure S2A in Supplementary Material). The number of CD8α^+^ spleen DCs (CD11c^hi^MHCII^hi^Sirpα^neg^CD24^pos^FLT3^pos^) was similar between WT and Nur77^−/−^ mice. Also in the LNs, the presence of resident (CD11c^hi^MHCII^+^) and migratory DCs (CD11c^+^MHCII^hi^) was comparable (Figure 2B; Figure S2B in Supplementary Material). Also in a transplantable autologous TH-MYCN 9464D mouse model of neuroblastoma (53), we did not observe differences in the presence of the different subsets of DCs in the spleen or (non)draining LN (Figures S3A,B in Supplementary Material). These data indicate that Nur77 is dispensable for DC development. Next, we tested the expression level of Nur77 in different DC subsets by analyzing DCs from the spleen and LNs from transgenic Nur77 reporter mice that express GFP upon activation of the Nur77 promoter. Nur77^GFP^ was clearly expressed in CD11b^+^ spleen DCs. The expression in CD8α^+^ spleen DCs was less well defined and consisted of a population expressing Nur77^GFP^ at a very low level and a population expressing Nur77^GFP^ to a similar level as the CD11b^+^ DCs (Figures 3A,B). Resident DCs of inguinal and axillary LN expressed clear levels of Nur77^GFP^, in contrast to significantly lower expression in migratory DCs of these LN (Figures 3C,D). Mice bearing a neuroblastoma tumor showed a similar Nur77^GFP^ expression pattern in DCs (Figures S3C,D in Supplementary Material) as in naïve mice. These data indicate that Nur77 expression does not have a major impact on the development and presence of different DC subsets in the spleen and LNs and that Nur77 is most abundantly expressed in CD11b^+^ spleen DCs and resident DCs of different LNs.

### Nur77-Deficient Murine DCs Have Altered Cytokine Production and T Cell Stimulatory Capacity

**Figure 4:**
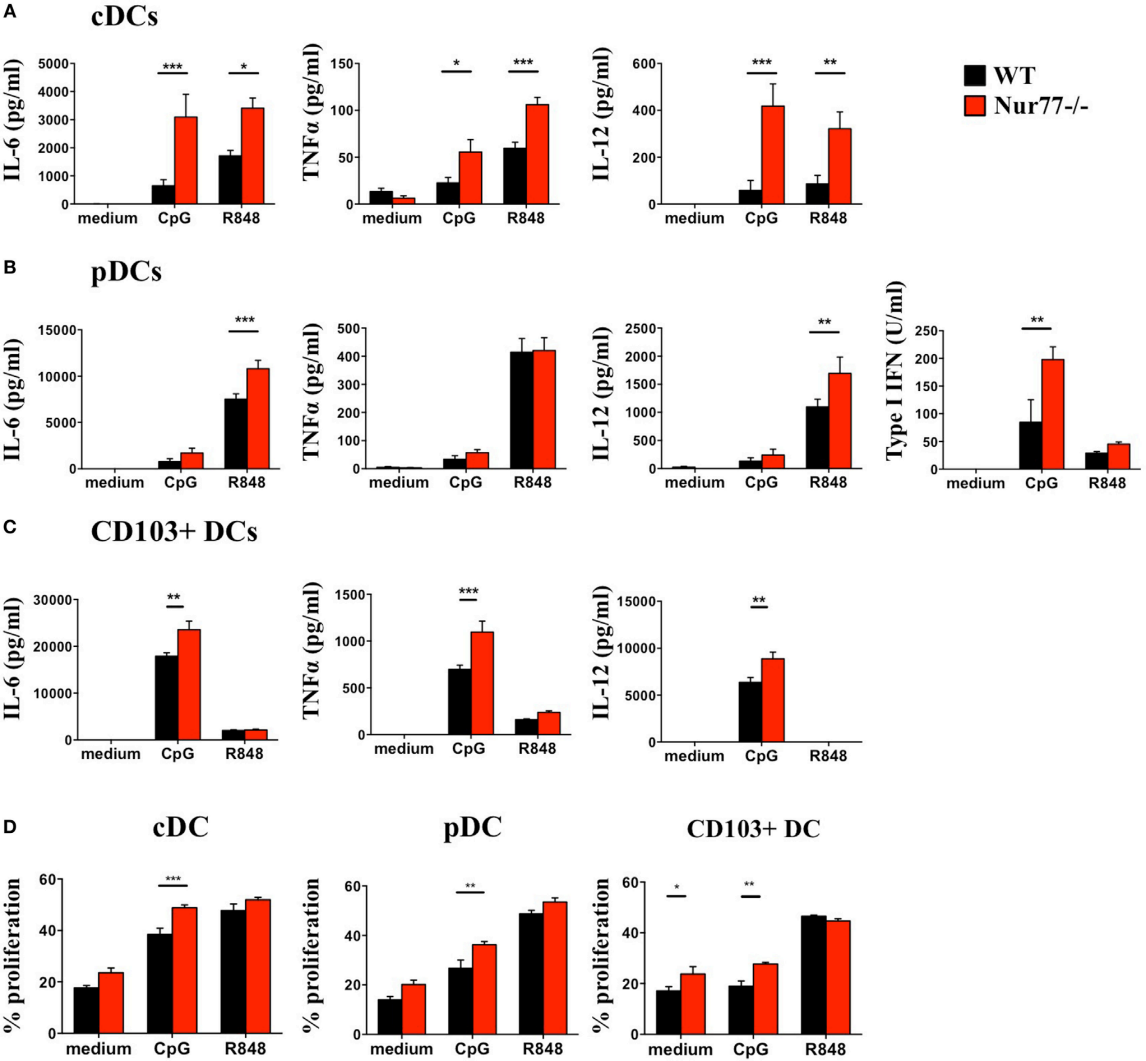
Cytokine production and T cell proliferation by murine Nur77^−/−^ dendritic cells (DCs) are increased compared with WT DCs. Conventional DCs (cDCs) **(A)** and plasmacytoid DCs (pDCs) **(B)** were sorted from FLT3L bone marrow cultures or CD103^+^ DCs **(C)** derived from FLT3L/GM-CSF cultures were stimulated with CpG or R848, cytokine production was measured with ELISA. T cell proliferation **(D)** was measured after coculture of CpG or R848 stimulated cDCs, pDCs, or CD103^+^ DCs with BalB/C T cells and was measured as indicated by CFSE dilution on day 3. Data are shown as the mean ± SEM, two-way ANOVA with Bonferroni posttest (*n* = 3–6 different mice) (*P < 0.05; **P < 0.01; and ***P < 0.001).

To assess the functional role of Nur77 in different murine DC subsets, we investigated cytokine production by murine Nur77^−/−^ BMDCs after stimulation with different inflammatory stimuli. We found that Nur77^−/−^ cDCs produced significantly more IL-6, TNFα, and IL-12 upon CpG and R848 stimulation (Figure 4A). Nur77^−/−^ pDCs showed increased production of IL-6 and IL-12 upon R848 stimulation, whereas TNFα production was not affected. After stimulation with CpG, type I IFN production was much higher in Nur77^−/−^ pDCs compared with WT pDCs (Figure 4B). CD103^+^ DCs showed a stronger response to CpG than to R848, revealing increased production of IL-6, TNFα, and IL-12 (Figure 4C). To rule out the possibility that the increase in cytokine production was (partly) mediated by enhanced TLR expression, we profiled TLR7 and TLR9 expression in these cells. TLR7 and TLR9 expression was similar in WT and Nur77^−/−^ cDCs, whereas TLR7 expression was reduced in Nur77^−/−^ pDCs (Figure S4 in Supplementary Material). In addition to cytokine production, we investigated the T-cell stimulatory capacities for Nur77-deficient DCs. To this end, control, CpG, or R848 stimulated DCs were added to an allogeneic MLR. All Nur77-deficient DC subsets were significantly more potent in inducing T cell proliferation than WT DCs upon stimulation with CpG (Figure 4D). These data indicate that Nur77 deficiency in DCs leads to enhanced cytokine production and subsequent increased T cell proliferation.

### Nur77 Expression and Function in Human DCs

**Figure 5:**
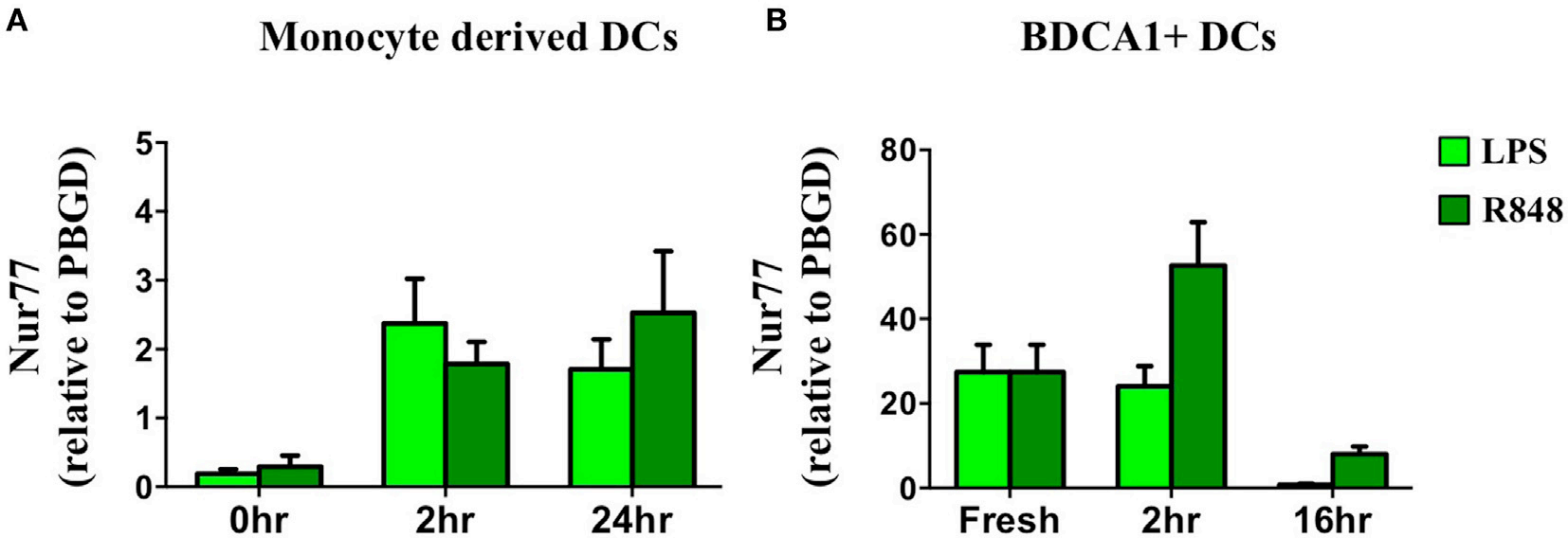
Nur77 mRNA expression in human dendritic cells (DCs). Human day 6 monocyte-derived dendritic cells **(A)** or freshly isolated BDCA1^+^ DCs **(B)** were stimulated for different time periods with LPS or R848. mRNA expression levels of Nur77 were detected by qPCR analysis. Expression levels shown are related to the housekeeping gene porphobilinogen deaminase (PBGD). Data shown are the mean ± SEM (*n* = 3–5 different donors).

In addition to defining its expression and function in murine DCs we profiled Nur77 mRNA expression in human moDCs after stimulation with LPS and R848. In accordance with murine DCs, human moDCs quickly upregulated Nur77 mRNA expression and the expression remained stable for 24 h after stimulation with either LPS or R848 (Figure 5A). To investigate Nur77 expression in freshly isolated BDCA1^+^ blood myeloid DCs, purified BDCA1^+^ DCs were stimulated for different time periods with LPS or R848 (Figure 5B). Compared with moDCs, freshly isolated BDCA1^+^ DCs had much higher expression levels of Nur77 expression under resting conditions. Stimulation with R848 led to a further increase of Nur77 expression, which diminished to lower levels 16 h after stimulation. These data indicate that in different subsets of human DCs Nur77 is expressed with varying expression levels.

### Human Nur77-Modified DCs Have Altered Cytokine Production and T Cell Stimulatory Capacity

**Figure 6:**
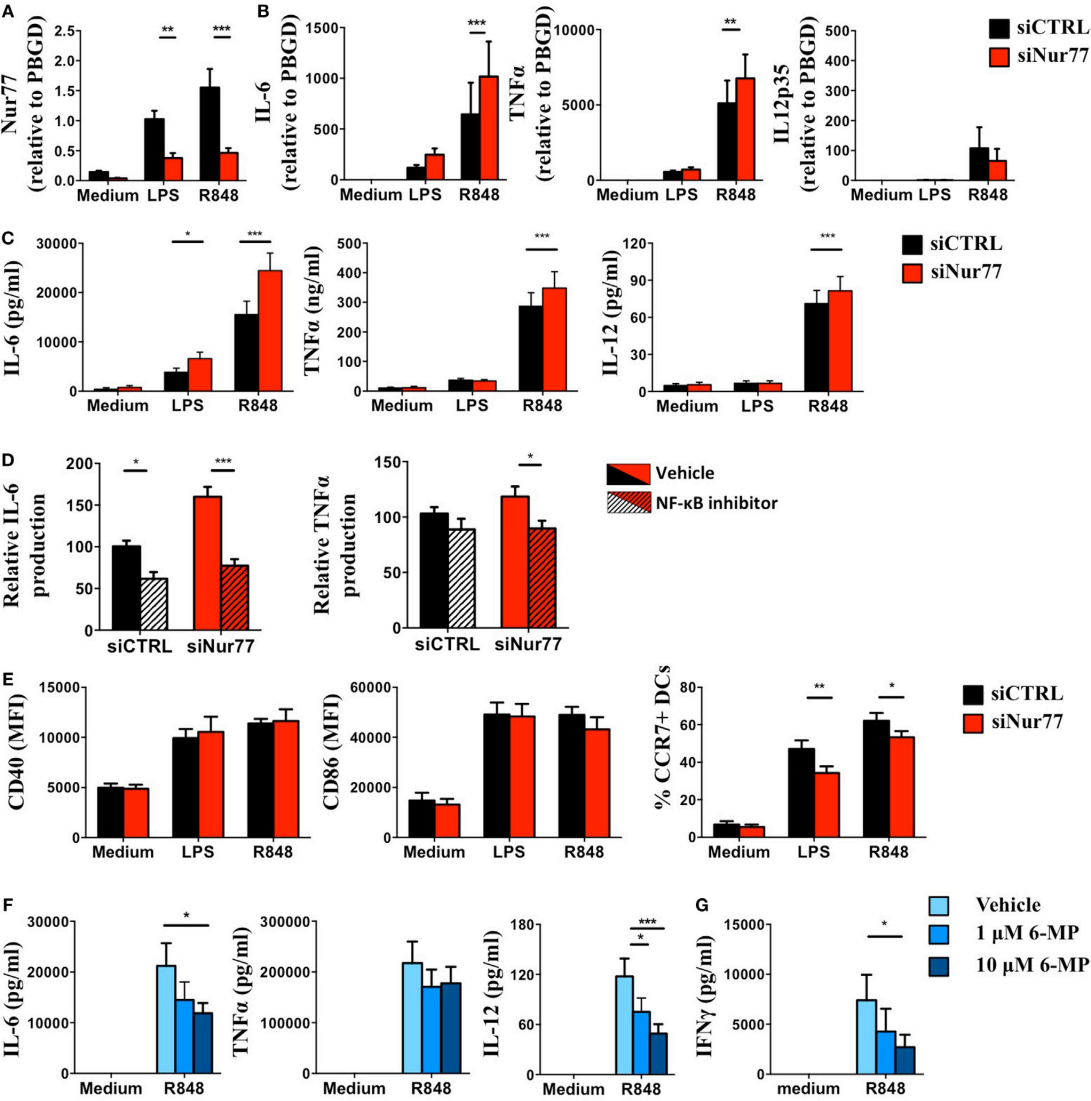
Knockdown or activation of Nur77 in human dendritic cells (DCs) alters DC function and T cell activation. Day 4 monocyte-derived dendritic cells (moDCs) were electroporated with a smartpool siRNA targeting Nur77 (siNur77) or a control siRNA (siCTRL). At day 6, cells were stimulated for 8 h with LPS, and Nur77 mRNA expression was detected by qPCR analysis **(A)**, cytokine mRNA expression after 8 h of stimulation was measured by qPCR analysis **(B)**, and cytokine levels were measured 24 h after stimulation with ELISA. **(C)** Electroporated moDCs were pretreated with Bay11-7082 and then stimulated with R848 for 24 h. Cytokine levels were measured by ELISA. **(D)** CD40, CD86, and CCR7 expression was determined by FACS analysis. **(E)** moDCs were pretreated with 6-mercaptopurine (6-MP) and then stimulated with TLRL for 24 h. Cytokine levels were measured by ELISA. **(F)** moDCs were pretreated with 6-MP and subsequently stimulated with R848, T cell stimulatory capacity was measured in an allogeneic mixed leukocyte reaction by measuring IFNγ production by ELISA **(G)**. Data shown are the mean ± SEM. Two-way ANOVA with Bonferroni posttest (*n* = 3–12 independent donors): *P < 0.05; **P < 0.01; and ***P < 0.001.

To test Nur77 function in human DCs, we silenced Nur77 expression in moDCs using a siRNA smartpool. Nur77 expression in moDCs decreased by 60–70% using siNur77 compared with control siRNA (siCTRL) (Figure 6A). These siNur77 targeted DCs had increased mRNA and protein expression of IL-6 and TNFα compared with siCTRL-treated DCs (Figures 6B,C), especially after R848 stimulation. Nur77-deficient DCs also showed enhanced IL-12 protein production. Profiling of TLR4, TLR7, and TLR8 expression (Figure S5 in Supplementary Material), revealed no change in TLR expression, indicating that the effect on cytokine production is not mediated *via* altered TLR expression. As NR4A family members have been reported to crosstalk with the NF-κB pathway (54), we investigated whether the enhanced cytokine production was dependent on NF-κB signaling. Blocking NF-κB signaling with the NF-κB inhibitor BAY11-7082 inhibited IL-6 and TNFα production in siNur77 DCs and siCTRL DCs to the same level (Figure 6D), indicating that the enhanced expression of IL-6 and TNFα was indeed dependent on NF-κB signaling. We next determined the expression of CD40, CD86, and CCR7 in siNur77 DCs. While siNur77 and siCTRL DCs show similar expression of the co-stimulatory markers CD40 and CD86, a significantly lower percentage of CCR7^+^ DCs were present in siNur77 DCs (Figure 6E). To further substantiate these data, we treated DCs with 6-MP, an activator of Nur77 (55–59). Treating DCs with 6-MP before stimulation with R848, led to a dose-dependent decrease of IL-6 and IL-12 production, while TNFα levels were not altered (Figure 6F). No effect of 6-MP on cell viability could be detected (Figure S6 in Supplementary Material). In line with decreased IL-6 and IL-12 production, DCs pretreated with 6-MP were less capable of inducing IFNγ production by T cells in an allogeneic MLR (Figure 6G). These data show that human Nur77-modified moDCs have altered NF-κB-dependent inflammatory responses that are important in inducing T cell activation.

## Discussion

Nuclear receptors have been shown to play a critical role in immune cell function, including members of the NR4A subgroup. However, the expression and function of Nur77 in different DC subsets has not been studied so far. We now show that Nur77 is expressed in different human as well as murine DC subsets. Its expression is rapidly upregulated upon stimulation with different TLR ligands. Deficiency of Nur77 leads to enhanced NF-κB dependent cytokine production and T cell stimulatory capacity of DCs, while stimulation with the Nur77 activator 6-MP limits cytokine production by DCs and its capacity to stimulate allogeneic T cells.

Nur77 expression has been shown to be essential in the differentiation and survival of Ly-6C^−^ monocytes (34, 35), in the polarization of macrophages (37, 38, 60) and in the function and negative selection of T cells (31, 32). This NR is also expressed in infiltrating monocytes and monocyte-derived macrophages of the CNS that are important in experimental autoimmune encephalomyelitis (28) and in patrolling monocytes that control metastasis to the lung (61). We now show, in line with its classification as early response gene, that Nur77 expression is quickly upregulated in different human and murine DC subsets after stimulation with distinct TLR ligands *in vitro*. However, the expression levels in the different DC subsets and level of response toward diverse stimuli vary. We also found Nur77 expression in different subsets of DCs in the spleen and LNs directly in naïve and in tumor-bearing mice *ex vivo*. Expression was more pronounced in the CD11b^+^ DCs of the spleen compared with CD8α^+^ DCs and higher in the resident than in the migratory DCs of the LNs. Previously, it has been shown that Nur77 is not required for the differentiation of Ly-6C^hi^ monocytes into moDCs (36). We now also show that Nur77 deficiency does not have a major impact on the presence of different DC subsets in the spleen and LN at steady state conditions as well as mice bearing a neuroblastoma tumor. This confirms that in contrast to its expression in Ly-6C^−^ monocytes, Nur77 expression is dispensable for the development of spleen and LN DCs.

Although most studies have reported that Nur77 has an anti-inflammatory role in monocytes and macrophages (37, 38, 62), it has been shown that its overexpression in murine macrophages can lead to a pro-inflammatory response (63). Our data point towards an anti-inflammatory role in human and murine DC subsets. Nur77 deficiency in DCs leads to enhanced production of IL-6, TNFα, and IL-12 and subsequent enhanced T cell proliferation, while Nur77 activation leads to reduced IL-6 and IL-12 production and reduced T cell activation. It has been hypothesized that Nur77 acts to resolve inflammation in macrophages (38, 64) and based on our data we now suggest a similar role for Nur77 in DCs.

All NR4A family members, including Nur77, have been shown to modulate immune cell function *via* crosstalk to NF-κB (30, 38, 54, 65). Our data show that also in human DCs, Nur77 affects cytokine production by modulating the NF-κB pathway. It has been shown that Nur77 can affect the NF-κB pathway signaling in numerous ways (38, 63, 65–68). Besides modulating phosphorylation of p65 Ser536 and Ser529 in macrophages (38, 69), Nur77 has also been shown to directly interact with the p65 subunit of NF-κB (65, 66) and block p65 binding to DNA (65). Moreover, Nur77 can regulate TRAF6 auto-ubiquitination (67), important for NF-κB signal transduction (70–72). Future studies should reveal which mechanism underlies Nur77-mediated modulation of NF-κB signaling in DCs and whether different DC subsets or different inflammatory conditions involve specific ways of regulating NF-κB signaling.

While Nur77-deficient DCs show enhanced inflammatory responses, pretreating human DCs with 6-MP led to reduced inflammatory responses and a diminished capacity to induce IFNγ production by T cells in an allogeneic MLR. 6-MP is a nucleic acid analog and has been shown to enhance Nur77 transcriptional activity (55–59). Currently, it is being applied as an immunosuppressive drug for the treatment of several chronic inflammatory diseases such as inflammatory bowel disease, systemic lupus erythematosus, acute lymphoblastic leukemia of childhood, inflammatory myopathies, and rheumatoid arthritis and to prevent acute rejection in organ transplant patients (73–75). It has been shown that besides activating Nur77 (55, 56, 76) 6-MP can also activate the NR4A members Nurr1 (77) and NOR-1 (76) and inhibit the GTPase proteins Rac1 and Rac2 (78, 79). Therefore, the effect observed in moDCs may be a combined effect of 6-MP on the function of either of these proteins. In addition to 6-MP many other pharmacological compounds have been generated to modulate Nur77 function. Among them are different C-DIMs [synthetic 1,1-bis(3′-indolyl)-1-(substituted phenyl)methane analogs] (80), cytosporone B and its structural analogs (81, 82), and TMPA (ethyl 2-[2,3,4-trimethoxy-6-(1-octanoyl)phenyl]acetate) (83). They have been shown to regulate Nur77 function by modulating Nur77-dependent transactivation, influencing its expression levels, inducing nuclear export of Nur77 or affecting binding to other proteins (80–88). Many of these compounds have, as also shown for 6-MP, also Nur77-independent actions (85, 89, 90). In cancer cells, neuronal cells, as well as different immune cells, it has been shown that Nur77 function depends on tissue context, subcellular localization, external stimuli, protein–protein interactions, or post-translational modifications (22–26, 31, 32, 34, 35, 37, 38, 60). How Nur77 function in DCs is exactly regulated upon specific immune stimuli and whether that is different in different DC subsets is currently unknown. Future studies should aim at fully elucidating whether specific stimuli in different subsets of DCs and under specific (pathological) conditions affect Nur77 activation and thereby modulate DC function. More knowledge regarding the exact mechanism(s) of Nur77 activation in DCs will help to choose the best pharmacological compound targeting specific actions of Nur77 in DCs. This will not only be important in optimizing current DC-based immunotherapies but also when more generally targeting Nur77 in different cell types and pathological conditions.

Interestingly, in tumor cells, the natural steroid Dendrogenin A has been shown to stimulate expression of Nur77 *via* binding to LXRβ and induce lethal autophagy (91, 92), opening up new perspectives for cancer treatment (93). Moreover, it has been shown that Dendrogenin A, in addition to inducing growth control and improve overall survival in mice, also induces immune cell infiltration, including DCs, in the tumor (94). As LXR has been shown to affect DC differentiation, maturation and migration (95–101), it is tempting to speculate that part of these effects are mediated *via* regulation of Nur77 expression, especially when DCs are stimulated with Dendrogenin A.

One striking observation is that the percentage of CCR7 expressing human DCs was decreased in siNur77 treated moDCs. Interestingly, another member of the NR4A subfamily, NOR-1, has been shown to affect CCR7-dependent murine CD103^+^ DC migration from tissues to LNs *in vivo* (42). Nevertheless, we did not observe a similar effect on CCR7 expression in *in vitro* cultured murine CD103^+^ DCs (data not shown). In agreement with Park et al., we did not find differences in the number of migratory murine DCs present in the LN in Nur77^−/−^ mice compared with WT mice, suggesting a less pronounced role for Nur77 in CCR7-dependent DC migration in mice. However, since NR4A family members are highly homologous proteins and can have redundant functions (102–104), it is also possible that the absence of Nur77 is compensated by NOR-1 in murine DCs.

Given that Nur77 modifies DC function with altered inflammatory responses, Nur77 may be an interesting therapeutic target to either boost or diminish the activation status of DCs in DC-based vaccination strategies in cancer or treatment of autoimmune diseases, respectively.

## Ethics Statement

All animal experiments were approved by the Radboud University’s Animal Welfare Body (AWB) (*Instantie voor Dierenwelzijn IvD*) and the Animal Experiment Committee (*DierExperimentenCommissie, RUDEC*) that is recognized by the CCD (Central Authority for Scientific Procedures on Animals). The experiments were performed according to institutional, national, and European guidelines as stipulated in the *Wet op de dierproeven* (WOD) and in the *Dierproevenbesluit*. All experiments involving human material were carried out after obtaining written informed consent from all subjects as per the Declaration of Helsinki. The study was approved by the Institutional Review Board of the Radboud University Nijmegen Medical Center, Commissie Mensgebonden Onderzoek.

## Author Contributions

NT-K and MA planned and performed experiments. EK-R and ML performed experiments. HI generated and provided mice. NT-K, CV, and MA contributed to the interpretation of the data. MA wrote the manuscript. NT-K, EK-R, ML, HI, and CV contributed to the review of the manuscript. MA designed the study.

## Conflict of Interest Statement

The authors declare that the research was conducted in the absence of any commercial or financial relationships that could be construed as a potential conflict of interest.

## References

[1] Granucci F Foti M Ricciardi-Castagnoli P. Dendritic cell biology. Adv Immunol (2005) 88:193–233. 10.1016/S0065-2776(05)88006-X 16227091

[2] Adema GJ. Dendritic cells from bench to bedside and back. Immunol Lett (2009) 122(2):128–30. 10.1016/j.imlet.2008.11.017 19121337

[3] Merad M Sathe P Helft J Miller J Mortha A. The dendritic cell lineage: ontogeny and function of dendritic cells and their subsets in the steady state and the inflamed setting. Annu Rev Immunol (2013) 31:563–604. 10.1146/annurev-immunol-020711-074950 23516985 PMC3853342

[4] Granot T Senda T Carpenter DJ Matsuoka N Weiner J Gordon CL Dendritic cells display subset and tissue-specific maturation dynamics over human life. Immunity (2017) 46(3):504–15. 10.1016/j.immuni.2017.02.019 28329707 PMC5415308

[5] Pakalniskyte D Schraml BU. Tissue-specific diversity and functions of conventional dendritic cells. Adv Immunol (2017) 134:89–135. 10.1016/bs.ai.2017.01.003 28413024

[6] Jacobs JF Punt CJ Lesterhuis WJ Sutmuller RP Brouwer HM Scharenborg NM Dendritic cell vaccination in combination with anti-CD25 monoclonal antibody treatment: a phase I/II study in metastatic melanoma patients. Clin Cancer Res (2010) 16(20):5067–78. 10.1158/1078-0432.CCR-10-1757 20736326

[7] Strioga MM Felzmann T Powell DJ Jr Ostapenko V Dobrovolskiene NT Matuskova M Therapeutic dendritic cell-based cancer vaccines: the state of the art. Crit Rev Immunol (2013) 33(6):489–547. 10.1615/CritRevImmunol.2013008033 24266347

[8] Aarntzen EH De Vries IJ Lesterhuis WJ Schuurhuis D Jacobs JF Bol K Targeting CD4(+) T-helper cells improves the induction of antitumor responses in dendritic cell-based vaccination. Cancer Res (2013) 73(1):19–29. 10.1158/0008-5472.CAN-12-1127 23087058

[9] Aarntzen EH Schreibelt G Bol K Lesterhuis WJ Croockewit AJ de Wilt JH Vaccination with mRNA-electroporated dendritic cells induces robust tumor antigen-specific CD4+ and CD8+ T cells responses in stage III and IV melanoma patients. Clin Cancer Res (2012) 18(19):5460–70. 10.1158/1078-0432.CCR-11-3368 22896657

[10] Aarntzen EH Srinivas M Schreibelt G Heerschap A Punt CJ Figdor CG Reducing cell number improves the homing of dendritic cells to lymph nodes upon intradermal vaccination. Oncoimmunology (2013) 2(7):e24661. 10.4161/onci.24661 24073362 PMC3782158

[11] Lo J Clare-Salzler MJ. Dendritic cell subsets and type I diabetes: focus upon DC-based therapy. Autoimmun Rev (2006) 5(6):419–23. 10.1016/j.autrev.2005.12.001 16890897

[12] Dannull J Haley NR Archer G Nair S Boczkowski D Harper M Melanoma immunotherapy using mature DCs expressing the constitutive proteasome. J Clin Invest (2013) 123(7):3135–45. 10.1172/JCI67544 23934126 PMC3696565

[13] Sabado RL Bhardwaj N. Directing dendritic cell immunotherapy towards successful cancer treatment. Immunotherapy (2010) 2(1):37–56. 10.2217/imt.09.43 20473346 PMC2867472

[14] Hansen M Met O Svane IM Andersen MH. Cellular based cancer vaccines: type 1 polarization of dendritic cells. Curr Med Chem (2012) 19(25):4239–46. 10.2174/092986712802884213 22834814

[15] Garg AD Vara Perez M Schaaf M Agostinis P Zitvogel L Kroemer G Trial watch: dendritic cell-based anticancer immunotherapy. Oncoimmunology (2017) 6(7):e1328341. 10.1080/2162402X.2017.1328341 28811970 PMC5543823

[16] Chow EK Razani B Cheng G. Innate immune system regulation of nuclear hormone receptors in metabolic diseases. J Leukoc Biol (2007) 82(2):187–95. 10.1189/jlb.1206741 17314330

[17] Ogawa S Lozach J Benner C Pascual G Tangirala RK Westin S Molecular determinants of crosstalk between nuclear receptors and toll-like receptors. Cell (2005) 122(5):707–21. 10.1016/j.cell.2005.06.029 16143103 PMC1430687

[18] Chute JP Ross JR McDonnell DP. Minireview: nuclear receptors, hematopoiesis, and stem cells. Mol Endocrinol (2010) 24(1):1–10. 10.1210/me.2009-0332 19934345 PMC2802897

[19] Hontelez S Karthaus N Looman MW Ansems M Adema GJ. DC-SCRIPT regulates glucocorticoid receptor function and expression of its target GILZ in dendritic cells. J Immunol (2013) 190(7):3172–9. 10.4049/jimmunol.1201776 23440419

[20] Karthaus N van Spriel AB Looman MW Chen S Spilgies LM Lieben L Vitamin D controls murine and human plasmacytoid dendritic cell function. J Invest Dermatol (2014) 134(5):1255–64. 10.1038/jid.2013.501 24352045

[21] Hamers AA Hanna RN Nowyhed H Hedrick CC de Vries CJ. NR4A nuclear receptors in immunity and atherosclerosis. Curr Opin Lipidol (2013) 24(5):381–5. 10.1097/MOL.0b013e3283643eac 24005216 PMC4709022

[22] Beard JA Tenga A Chen T. The interplay of NR4A receptors and the oncogene-tumor suppressor networks in cancer. Cell Signal (2015) 27(2):257–66. 10.1016/j.cellsig.2014.11.009 25446259 PMC4276441

[23] Wei X Gao H Zou J Liu X Chen D Liao J Contra-directional coupling of Nur77 and Nurr1 in neurodegeneration: a novel mechanism for memantine-induced anti-inflammation and anti-mitochondrial impairment. Mol Neurobiol (2016) 53(9):5876–92. 10.1007/s12035-015-9477-7 26497037

[24] Gao H Chen Z Fu Y Yang X Weng R Wang R Nur77 exacerbates PC12 cellular injury in vitro by aggravating mitochondrial impairment and endoplasmic reticulum stress. Sci Rep (2016) 6:34403. 10.1038/srep34403 27679973 PMC5041156

[25] Montarolo F Perga S Martire S Navone DN Marchet A Leotta D Altered NR4A subfamily gene expression level in peripheral blood of parkinson’s and alzheimer’s disease patients. Neurotox Res (2016) 30(3):338–44. 10.1007/s12640-016-9626-4 27159982

[26] Zou J Chen Z Wei X Chen Z Fu Y Yang X Cystatin C as a potential therapeutic mediator against Parkinson’s disease via VEGF-induced angiogenesis and enhanced neuronal autophagy in neurovascular units. Cell Death Dis (2017) 8(6):e2854. 10.1038/cddis.2017.240 28569795 PMC5520899

[27] Rothe T Ipseiz N Faas M Lang S Perez-Branguli F Metzger D The nuclear receptor Nr4a1 acts as a microglia rheostat and serves as a therapeutic target in autoimmune-driven central nervous system inflammation. J Immunol (2017) 198(10):3878–85. 10.4049/jimmunol.1600638 28411187 PMC5798579

[28] Shaked I Hanna RN Shaked H Chodaczek G Nowyhed HN Tweet G Transcription factor Nr4a1 couples sympathetic and inflammatory cues in CNS-recruited macrophages to limit neuroinflammation. Nat Immunol (2015) 16(12):1228–34. 10.1038/ni.3321 26523867 PMC4833087

[29] Maxwell MA Muscat GE. The NR4A subgroup: immediate early response genes with pleiotropic physiological roles. Nucl Recept Signal (2006) 4:e002. 10.1621/nrs.04002 16604165 PMC1402209

[30] McMorrow JP Murphy EP. Inflammation: a role for NR4A orphan nuclear receptors? Biochem Soc Trans (2011) 39(2):688–93. 10.1042/BST0390688 21428963

[31] Fassett MS Jiang W D’Alise AM Mathis D Benoist C. Nuclear receptor Nr4a1 modulates both regulatory T-cell (Treg) differentiation and clonal deletion. Proc Natl Acad Sci U S A (2012) 109(10):3891–6. 10.1073/pnas.1200090109 22345564 PMC3309794

[32] Zhou T Cheng J Yang P Wang Z Liu C Su X Inhibition of Nur77/Nurr1 leads to inefficient clonal deletion of self-reactive T cells. J Exp Med (1996) 183(4):1879–92. 10.1084/jem.183.4.1879 8666944 PMC2192482

[33] Sekiya T Kashiwagi I Yoshida R Fukaya T Morita R Kimura A Nr4a receptors are essential for thymic regulatory T cell development and immune homeostasis. Nat Immunol (2013) 14(3):230–7. 10.1038/ni.2520 23334790

[34] Hanna RN Carlin LM Hubbeling HG Nackiewicz D Green AM Punt JA The transcription factor NR4A1 (Nur77) controls bone marrow differentiation and the survival of Ly6C-monocytes. Nat Immunol (2011) 12(8):778–85. 10.1038/ni.2063 21725321 PMC3324395

[35] Carlin LM Stamatiades EG Auffray C Hanna RN Glover L Vizcay-Barrena G Nr4a1-dependent Ly6C(low) monocytes monitor endothelial cells and orchestrate their disposal. Cell (2013) 153(2):362–75. 10.1016/j.cell.2013.03.010 23582326 PMC3898614

[36] Briseno CG Haldar M Kretzer NM Wu X Theisen DJ Kc W Distinct transcriptional programs control cross-priming in classical and monocyte-derived dendritic cells. Cell Rep (2016) 15(11):2462–74. 10.1016/j.celrep.2016.05.025 27264183 PMC4941620

[37] Bonta PI van Tiel CM Vos M Pols TW van Thienen JV Ferreira V Nuclear receptors Nur77, Nurr1, and NOR-1 expressed in atherosclerotic lesion macrophages reduce lipid loading and inflammatory responses. Arterioscler Thromb Vasc Biol (2006) 26(10):2288–94. 10.1161/01.ATV.0000238346.84458.5d 16873729

[38] Hanna RN Shaked I Hubbeling HG Punt JA Wu R Herrley E NR4A1 (Nur77) deletion polarizes macrophages toward an inflammatory phenotype and increases atherosclerosis. Circ Res (2012) 110(3):416–27. 10.1161/CIRCRESAHA.111.253377 22194622 PMC3309661

[39] Wang T Jiang Q Chan C Gorski KS McCadden E Kardian D Inhibition of activation-induced death of dendritic cells and enhancement of vaccine efficacy via blockade of MINOR. Blood (2009) 113(13):2906–13. 10.1182/blood-2008-08-176354 19164597 PMC2662637

[40] Karthaus N Hontelez S Looman MW van Spriel AB Ansems M Adema GJ. Nuclear receptor expression patterns in murine plasmacytoid and conventional dendritic cells. Mol Immunol (2013) 55(3–4):409–17. 10.1016/j.molimm.2013.03.020 23597769

[41] Grajales-Reyes GE Iwata A Albring J Wu X Tussiwand R Kc W Batf3 maintains autoactivation of Irf8 for commitment of a CD8alpha(+) conventional DC clonogenic progenitor. Nat Immunol (2015) 16(7):708–17. 10.1038/ni.3197 26054719 PMC4507574

[42] Park K Mikulski Z Seo GY Andreyev AY Marcovecchio P Blatchley A The transcription factor NR4A3 controls CD103+ dendritic cell migration. J Clin Invest (2016) 126(12):4603–15. 10.1172/JCI87081 27820700 PMC5127666

[43] Saini A Mahajan S Gupta P. Nuclear receptor expression atlas in BMDCs: Nr4a2 restricts immunogenicity of BMDCs and impedes EAE. Eur J Immunol (2016) 46(8):1842–53. 10.1002/eji.201546229 27184189

[44] Nagaoka M Yashiro T Uchida Y Ando T Hara M Arai H The orphan nuclear receptor NR4A3 is involved in the function of dendritic cells. J Immunol (2017) 199(8):2958–67. 10.4049/jimmunol.1601911 28893954

[45] Sekiya T Kashiwagi I Inoue N Morita R Hori S Waldmann H The nuclear orphan receptor Nr4a2 induces Foxp3 and regulates differentiation of CD4+ T cells. Nat Commun (2011) 2:269. 10.1038/ncomms1272 21468021 PMC3104557

[46] Jiang Z Georgel P Du X Shamel L Sovath S Mudd S CD14 is required for MyD88-independent LPS signaling. Nat Immunol (2005) 6(6):565–70. 10.1038/ni1207 15895089

[47] Hontelez S Ansems M Karthaus N Zuidscherwoude M Looman MW Triantis V Dendritic cell-specific transcript: dendritic cell marker and regulator of TLR-induced cytokine production. J Immunol (2012) 189(1):138–45. 10.4049/jimmunol.1103709 22615205

[48] Sondergaard JN Poghosyan S Hontelez S Louche P Looman MW Ansems M DC-SCRIPT regulates IL-10 production in human dendritic cells by modulating NF-kappaBp65 activation. J Immunol (2015) 195(4):1498–505. 10.4049/jimmunol.1402924 26170389

[49] Livak KJ Schmittgen TD. Analysis of relative gene expression data using real-time quantitative PCR and the 2(-delta delta C(T)) method. Methods (2001) 25(4):402–8. 10.1006/meth.2001.1262 11846609

[50] Naik SH Proietto AI Wilson NS Dakic A Schnorrer P Fuchsberger M Cutting edge: generation of splenic CD8+ and CD8-dendritic cell equivalents in Fms-like tyrosine kinase 3 ligand bone marrow cultures. J Immunol (2005) 174(11):6592–7. 10.4049/jimmunol.174.11.6592 15905497

[51] Moran AE Holzapfel KL Xing Y Cunningham NR Maltzman JS Punt J T cell receptor signal strength in Treg and iNKT cell development demonstrated by a novel fluorescent reporter mouse. J Exp Med (2011) 208(6):1279–89. 10.1084/jem.20110308 21606508 PMC3173240

[52] Mayer CT Ghorbani P Nandan A Dudek M Arnold-Schrauf C Hesse C Selective and efficient generation of functional Batf3-dependent CD103+ dendritic cells from mouse bone marrow. Blood (2014) 124(20):3081–91. 10.1182/blood-2013-12-545772 25100743 PMC4260363

[53] Kroesen M Nierkens S Ansems M Wassink M Orentas RJ Boon L A transplantable TH-MYCN transgenic tumor model in C57Bl/6 mice for preclinical immunological studies in neuroblastoma. Int J Cancer (2014) 134(6):1335–45. 10.1002/ijc.28463 24038106

[54] Murphy EP Crean D Molecular interactions between NR4A orphan nuclear receptors and NF-kappaB are required for appropriate inflammatory responses and immune cell homeostasis. Biomolecules (2015) 5(3):1302–18. 10.3390/biom5031302 26131976 PMC4598753

[55] Yoo YG Na TY Yang WK Kim HJ Lee IK Kong G 6-Mercaptopurine, an activator of Nur77, enhances transcriptional activity of HIF-1alpha resulting in new vessel formation. Oncogene (2007) 26(26):3823–34. 10.1038/sj.onc.1210149 17146432

[56] Pires NM Pols TW de Vries MR van Tiel CM Bonta PI Vos M Activation of nuclear receptor Nur77 by 6-mercaptopurine protects against neointima formation. Circulation (2007) 115(4):493–500. 10.1161/CIRCULATIONAHA.106.626838 17242285

[57] Huang HY Chang HF Tsai MJ Chen JS Wang MJ 6-Mercaptopurine attenuates tumor necrosis factor-alpha production in microglia through Nur77-mediated transrepression and PI3K/Akt/mTOR signaling-mediated translational regulation. J Neuroinflammation (2016) 13(1):78 10.1186/s12974-016-0543-5 27075886 PMC4831152

[58] Qin Q Chen M Yi B You X Yang P Sun J. Orphan nuclear receptor Nur77 is a novel negative regulator of endothelin-1 expression in vascular endothelial cells. J Mol Cell Cardiol (2014) 77:20–8. 10.1016/j.yjmcc.2014.09.027 25284689 PMC4312239

[59] Shao Q Shen LH Hu LH Pu J Qi MY Li WQ Nuclear receptor Nur77 suppresses inflammatory response dependent on COX-2 in macrophages induced by oxLDL. J Mol Cell Cardiol (2010) 49(2):304–11. 10.1016/j.yjmcc.2010.03.023 20381497

[60] Hamers AA Argmann C Moerland PD Koenis DS Marinkovic G Sokolovic M Nur77-deficiency in bone marrow-derived macrophages modulates inflammatory responses, extracellular matrix homeostasis, phagocytosis and tolerance. BMC Genomics (2016) 17:162. 10.1186/s12864-016-2469-9 26932821 PMC4774191

[61] Hanna RN Cekic C Sag D Tacke R Thomas GD Nowyhed H Patrolling monocytes control tumor metastasis to the lung. Science (2015) 350(6263):985–90. 10.1126/science.aac9407 26494174 PMC4869713

[62] Hilgendorf I Gerhardt LM Tan TC Winter C Holderried TA Chousterman BG Ly-6Chigh monocytes depend on Nr4a1 to balance both inflammatory and reparative phases in the infarcted myocardium. Circ Res (2014) 114(10):1611–22. 10.1161/CIRCRESAHA.114.303204 24625784 PMC4017349

[63] Pei L Castrillo A Tontonoz P. Regulation of macrophage inflammatory gene expression by the orphan nuclear receptor Nur77. Mol Endocrinol (2006) 20(4):786–94. 10.1210/me.2005-0331 16339277

[64] Pei L Castrillo A Chen M Hoffmann A Tontonoz P. Induction of NR4A orphan nuclear receptor expression in macrophages in response to inflammatory stimuli. J Biol Chem (2005) 280(32):29256–62. 10.1074/jbc.M502606200 15964844

[65] Li L Liu Y Chen HZ Li FW Wu JF Zhang HK Impeding the interaction between Nur77 and p38 reduces LPS-induced inflammation. Nat Chem Biol (2015) 11(5):339–46. 10.1038/nchembio.1788 25822914

[66] Hong CY Park JH Ahn RS Im SY Choi HS Soh J Molecular mechanism of suppression of testicular steroidogenesis by proinflammatory cytokine tumor necrosis factor alpha. Mol Cell Biol (2004) 24(7):2593–604. 10.1128/MCB.24.7.2593-2604.2004 15024051 PMC371106

[67] Li XM Zhang S He XS Guo PD Lu XX Wang JR Nur77-mediated TRAF6 signalling protects against LPS-induced sepsis in mice. J Inflamm (Lond) (2016) 13:4. 10.1186/s12950-016-0112-9 26839514 PMC4735956

[68] Hamers AA van Dam L Teixeira Duarte JM Vos M Marinkovic G van Tiel CM Deficiency of nuclear receptor Nur77 aggravates mouse experimental colitis by increased NFkappaB activity in macrophages. PLoS One (2015) 10(8):e0133598 10.1371/journal.pone.0133598 26241646 PMC4524678

[69] Ipseiz N Uderhardt S Scholtysek C Steffen M Schabbauer G Bozec A The nuclear receptor Nr4a1 mediates anti-inflammatory effects of apoptotic cells. J Immunol (2014) 192(10):4852–8. 10.4049/jimmunol.1303377 24740500

[70] Kobayashi T Walsh PT Walsh MC Speirs KM Chiffoleau E King CG TRAF6 is a critical factor for dendritic cell maturation and development. Immunity (2003) 19(3):353–63. 10.1016/S1074-7613(03)00230-9 14499111

[71] Deng L Wang C Spencer E Yang L Braun A You J Activation of the IkappaB kinase complex by TRAF6 requires a dimeric ubiquitin-conjugating enzyme complex and a unique polyubiquitin chain. Cell (2000) 103(2):351–61. 10.1016/S0092-8674(00)00126-4 11057907

[72] Wang Y Tang Y Teng L Wu Y Zhao X Pei G. Association of beta-arrestin and TRAF6 negatively regulates toll-like receptor-interleukin 1 receptor signaling. Nat Immunol (2006) 7(2):139–47. 10.1038/ni1294 16378096

[73] Murray JE Merrill JP Harrison JH Wilson RE Dammin GJ Prolonged survival of human-kidney homografts by immunosuppressive drug therapy. N Engl J Med (1963) 268:1315–23. 10.1056/NEJM196306132682401 13936775

[74] Kornbluth A George J Sachar DB. Immunosuppressive drugs in Crohn’s disease. Gastroenterologist (1994) 2(3):239–46. 7987622

[75] Aarbakke J Janka-Schaub G Elion GB Thiopurine biology and pharmacology. Trends Pharmacol Sci (1997) 18(1):3–7. 10.1016/S0165-6147(96)01007-3 9114722

[76] Wansa KD Harris JM Yan G Ordentlich P Muscat GE. The AF-1 domain of the orphan nuclear receptor NOR-1 mediates trans-activation, coactivator recruitment, and activation by the purine anti-metabolite 6-mercaptopurine. J Biol Chem (2003) 278(27):24776–90. 10.1074/jbc.M300088200 12709428

[77] Ordentlich P Yan Y Zhou S Heyman RA. Identification of the antineoplastic agent 6-mercaptopurine as an activator of the orphan nuclear hormone receptor Nurr1. J Biol Chem (2003) 278(27):24791–9. 10.1074/jbc.M302167200 12709433

[78] Marinkovic G Hamers AA de Vries CJ de Waard V. 6-Mercaptopurine reduces macrophage activation and gut epithelium proliferation through inhibition of GTPase Rac1. Inflamm Bowel Dis (2014) 20(9):1487–95. 10.1097/MIB.0000000000000122 25029617

[79] Marinkovic G Kroon J Hoogenboezem M Hoeben KA Ruiter MS Kurakula K Inhibition of GTPase Rac1 in endothelium by 6-mercaptopurine results in immunosuppression in nonimmune cells: new target for an old drug. J Immunol (2014) 192(9):4370–8. 10.4049/jimmunol.1302527 24670805

[80] Lee SO Li X Hedrick E Jin UH Tjalkens RB Backos DS Diindolylmethane analogs bind NR4A1 and are NR4A1 antagonists in colon cancer cells. Mol Endocrinol (2014) 28(10):1729–39. 10.1210/me.2014-1102 25099012 PMC4179635

[81] Zhan Y Du X Chen H Liu J Zhao B Huang D Cytosporone B is an agonist for nuclear orphan receptor Nur77. Nat Chem Biol (2008) 4(9):548–56. 10.1038/nchembio.106 18690216

[82] Liu JJ Zeng HN Zhang LR Zhan YY Chen Y Wang Y A unique pharmacophore for activation of the nuclear orphan receptor Nur77 in vivo and in vitro. Cancer Res (2010) 70(9):3628–37. 10.1158/0008-5472.CAN-09-3160 20388790

[83] Zhan YY Chen Y Zhang Q Zhuang JJ Tian M Chen HZ The orphan nuclear receptor Nur77 regulates LKB1 localization and activates AMPK. Nat Chem Biol (2012) 8(11):897–904. 10.1038/nchembio.1069 22983157

[84] Chintharlapalli S Burghardt R Papineni S Ramaiah S Yoon K Safe S Activation of Nur77 by selected 1,1-Bis(3’-indolyl)-1-(p-substituted phenyl)methanes induces apoptosis through nuclear pathways. J Biol Chem (2005) 280(26):24903–14. 10.1074/jbc.M500107200 15871945

[85] Cho SD Lei P Abdelrahim M Yoon K Liu S Guo J 1,1-bis(3’-indolyl)-1-(p-methoxyphenyl)methane activates Nur77-independent proapoptotic responses in colon cancer cells. Mol Carcinog (2008) 47(4):252–63. 10.1002/mc.20378 17957723

[86] Safe S Jin UH Morpurgo B Abudayyeh A Singh M Tjalkens RB. Nuclear receptor 4A (NR4A) family – orphans no more. J Steroid Biochem Mol Biol (2016) 157:48–60. 10.1016/j.jsbmb.2015.04.016 25917081 PMC4618773

[87] Yoon K Lee SO Cho SD Kim K Khan S Safe S. Activation of nuclear TR3 (NR4A1) by a diindolylmethane analog induces apoptosis and proapoptotic genes in pancreatic cancer cells and tumors. Carcinogenesis (2011) 32(6):836–42. 10.1093/carcin/bgr040 21362629 PMC3106434

[88] Lee SO Li X Khan S Safe S. Targeting NR4A1 (TR3) in cancer cells and tumors. Expert Opin Ther Targets (2011) 15(2):195–206. 10.1517/14728222.2011.547481 21204731 PMC4407471

[89] Dawson MI Ye M Cao X Farhana L Hu QY Zhao Y Derivation of a retinoid X receptor scaffold from peroxisome proliferator-activated receptor gamma ligand 1-Di(1H-indol-3-yl)methyl-4-trifluoromethylbenzene. ChemMedChem (2009) 4(7):1106–19. 10.1002/cmdc.200800447 19378296 PMC3031428

[90] Cho SD Yoon K Chintharlapalli S Abdelrahim M Lei P Hamilton S Nur77 agonists induce proapoptotic genes and responses in colon cancer cells through nuclear receptor-dependent and nuclear receptor-independent pathways. Cancer Res (2007) 67(2):674–83. 10.1158/0008-5472.CAN-06-2907 17234778

[91] Segala G David M de Medina P Poirot MC Serhan N Vergez F Dendrogenin A drives LXR to trigger lethal autophagy in cancers. Nat Commun (2017) 8(1):1903. 10.1038/s41467-017-01948-9 29199269 PMC5712521

[92] Poirot M Silvente-Poirot S. The tumor-suppressor cholesterol metabolite, dendrogenin A, is a new class of LXR modulator activating lethal autophagy in cancers. Biochem Pharmacol (2018) 153:75–81. 10.1016/j.bcp.2018.01.046 29409832

[93] Silvente-Poirot S Segala G Poirot MC Poirot M. Ligand-dependent transcriptional induction of lethal autophagy: a new perspective for cancer treatment. Autophagy (2018) 14(3):555–7. 10.1080/15548627.2018.1425059 29368971 PMC5915042

[94] de Medina P Paillasse MR Segala G Voisin M Mhamdi L Dalenc F Dendrogenin A arises from cholesterol and histamine metabolism and shows cell differentiation and anti-tumour properties. Nat Commun (2013) 4:1840. 10.1038/ncomms2835 23673625 PMC3674249

[95] Zhong L Yang Q Xie W Zhou J. Liver X receptor regulates mouse GM-CSF-derived dendritic cell differentiation in vitro. Mol Immunol (2014) 60(1):32–43. 10.1016/j.molimm.2014.03.006 24747959

[96] Canavan M McCarthy C Larbi NB Dowling JK Collins L O’Sullivan F Activation of liver X receptor suppresses the production of the IL-12 family of cytokines by blocking nuclear translocation of NF-kappaBp50. Innate Immun (2014) 20(7):675–87. 10.1177/1753425913501915 24045337

[97] Geyeregger R Zeyda M Bauer W Kriehuber E Saemann MD Zlabinger GJ Liver X receptors regulate dendritic cell phenotype and function through blocked induction of the actin-bundling protein fascin. Blood (2007) 109(10):4288–95. 10.1182/blood-2006-08-043422 17255360

[98] Torocsik D Barath M Benko S Szeles L Dezso B Poliska S Activation of liver X receptor sensitizes human dendritic cells to inflammatory stimuli. J Immunol (2010) 184(10):5456–65. 10.4049/jimmunol.0902399 20410489

[99] Villablanca EJ Raccosta L Zhou D Fontana R Maggioni D Negro A Tumor-mediated liver X receptor-alpha activation inhibits CC chemokine receptor-7 expression on dendritic cells and dampens antitumor responses. Nat Med (2010) 16(1):98–105. 10.1038/nm.2074 20037595

[100] Beceiro S Pap A Czimmerer Z Sallam T Guillen JA Gallardo G LXR nuclear receptors are transcriptional regulators of dendritic cell chemotaxis. Mol Cell Biol (2018) 38(10):e00534–17. 10.1128/MCB.00534-17 29507185 PMC5954201

[101] Bruckner M Dickel D Singer E Legler DF Converse regulation of CCR7-driven human dendritic cell migration by prostaglandin E(2) and liver X receptor activation. Eur J Immunol (2012) 42(11):2949–58. 10.1002/eji.201242523 22890791

[102] Cheng LE Chan FK Cado D Winoto A. Functional redundancy of the Nur77 and Nor-1 orphan steroid receptors in T-cell apoptosis. EMBO J (1997) 16(8):1865–75. 10.1093/emboj/16.8.1865 9155013 PMC1169790

[103] Maira M Martens C Philips A Drouin J. Heterodimerization between members of the Nur subfamily of orphan nuclear receptors as a novel mechanism for gene activation. Mol Cell Biol (1999) 19(11):7549–57. 10.1128/MCB.19.11.7549 10523643 PMC84765

[104] Tontonoz P Cortez-Toledo O Wroblewski K Hong C Lim L Carranza R The orphan nuclear receptor Nur77 is a determinant of myofiber size and muscle mass in mice. Mol Cell Biol (2015) 35(7):1125–38. 10.1128/MCB.00715-14 25605333 PMC4355536

