# Supplementary material for: Nuclear Receptor Nur77 Deficiency Alters Dendritic Cell Function
Source: Front Immunol. 2018 Aug 3;9:1797. doi: 10.3389/fimmu.2018.01797 (PMC6085422; doi:10.3389/fimmu.2018.01797)
Supplement: Supplementary file 1 [file Data_Sheet_1.PDF]

Karthaus et al., Supplementary Table 1

Overview of FACS antibodies used

Target	Clone	Company (Cat. No.)
**murine CD172 (Brilliant Violet 510)**	P84	BD bioscience (740159)
**murine I-A/I-E (Brilliant Violet 510)**	M5/114.15.2	Sony Biotechnology (1138175)
**murine CD135 (Brilliant Violet 421)**	A2F10.1	BD bioscience (562898)
**murine B220 (CD45R) (FITC)**	RA3-6B2	Sony Biotechnology (1116030)
**murine CD11c (Alexa Fluor 488)**	N418	biolegend (117311)
**murine CD24 (Alexa Fluor 488)**	M1/69	Sony Biotechnology (1109080)
**murine Ly-6G (FITC)**	1A8	Sony Biotechnology (1238030)
**murine IgG1 Isotype ctrl (FITC)**	MOPC-21	Sony Biotechnology (2600540)
**murine SiglecH (FITC)**	eBio440c	eBioscience (11-0333-82)
**human CD40 (Pe)**	MAB89	Beckman Coulter (IM1936U)
**murine CD370 (Clec9A) (Pe)**	7H11	Miltenyi Biotec (130-102-362)
**murine I-A/I-E (PE)**	M5/114.15.2	Sony Biotechnology (1138040)
**murine IgG1 Isotype ctrl (PE)**	MOPC-21	Sony Biotechnology (2600560)
**murine B220 (CD45R) (PerCP)**	RA3-6B2	biolegend (103234)
**murine CD11b (PerCP)**	M1/70	Sony Biotechnology (1106150)
**human CD86 (PE-Cy7)**	Clone 2331 (FUN-1)	BD Bioscience (561128)
**murine CD103 (PE-Cy7)**	2E 7	Sony Biotechnology (1207130)
**murine CD115 (PE-Cy7)**	AFS98	eBioscience (25-1152-80)
**murine IgG1 Isotype ctrl (PE-Cy7)**	MOPC-21	Sony Biotechnology (2600630)
**murine CD11c (APC)**	N418	Sony Biotechnology (1186550)
**human CD197 (CCR7) (APC)**	FR 11-11E8	Miltenyi Biotec (130-093-624)
**murine siglecH (Alexa Fluor 647)**	440c	eBioscience (51-0333-82)
**murine CD4 (APC/Cy7)**	RM4-5	Sony Biotechnology (1102630)

Karthaus et al., Supplementary Table 2

Overview of qPCR primers used

	Forward	***Reverse***
**murine Nur77**	*5'ACGGTCCCTGCACAGCTT*	*5'ATGCGATTCTGCAGCTCTTC*
**human Nur77**	*5’CTGCCAATCTCCTCACTT*	*5’GATGACCTCCAGAGAACC*
**human TLR1**	*5’TAACAAAGGCATATTGGG*	*5’GGAACGTGGATGAGACCGTTT*
**human TLR3**	*5’CAAACACAAGCATTCGGAATCTG*	*5’AAGGAATCGTTACCAACCACATT*
**human TLR4**	*5’GGCATGCCTGTGCTGAGTT*	*5’CTGCTACAACAGATACTACAAGCACACT*
**human TLR7**	*5’TGCCATCAAGAAAGTTGATGCT*	*5’GGAATGTAGAGGTCTGGTTGAAGAG*
**human TLR8**	*5’CGGAATGAAAAATTAGAACAACAGAA*	*5’GAACCAGATATTAGCAGGAAAATGC*
**human TLR9**	*5’TGAAGACTTCAGGCCCAACTG*	*5’TGCACGGTCACCAGGTTGT*

Karthaus et al., Supplementary Figure 1

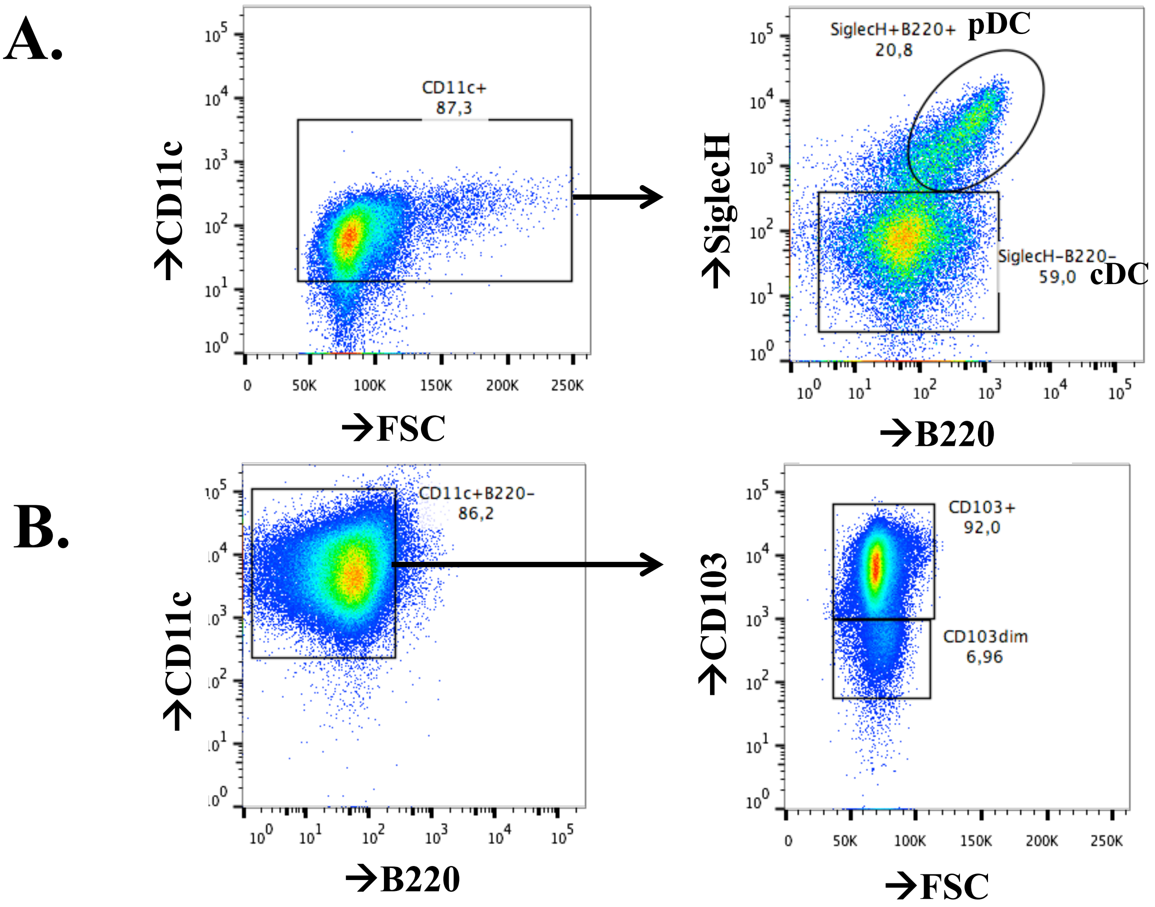

Supplementary figure 1. Gating strategy of BM derived murine DCs. A) cDCs and pDCs were generated by culturing BMDCs with FLT3L for 8-10 days. cDCs are identified as CD11c+B220-SiglecH- cells and pDCs as CD11c+B220+SiglecH+ cells **B)** CD103+ DCs are generated by culturing BMDCs with FLT3L and GM-CSF for 14 days and are identified as CD11c+B220-CD103+ cells

Karthaus et al., Supplementary Figure 2

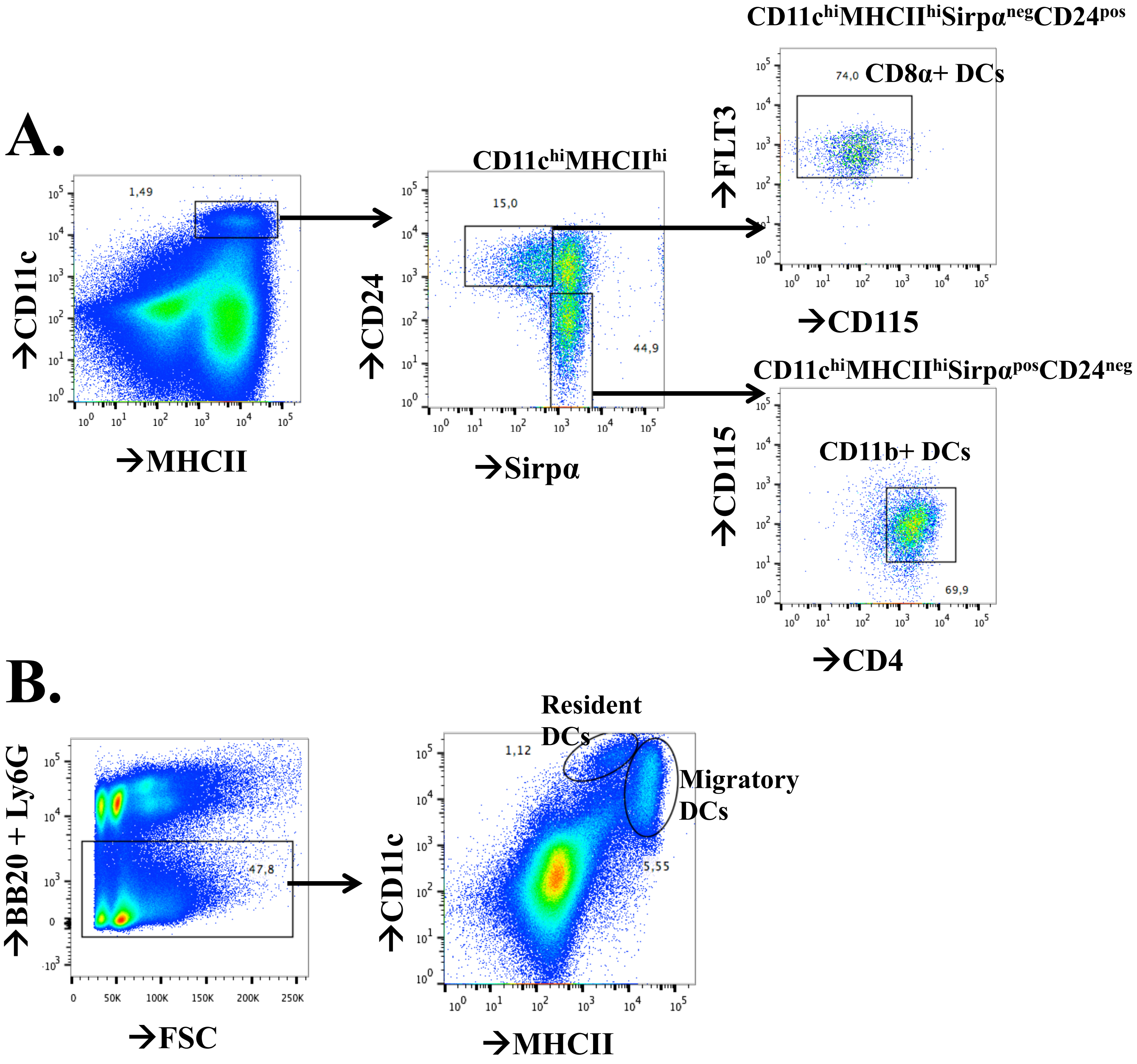

Supplementary figure 2. Gating strategy of splenic and lymph node DCs A) Single murine spleen cells were analyzed by flowcytometry according to the indicated strategy for the presence of CD8α+ DCs and CD11b+ DCs **B)** Single murine lymph node cells were analyzed by flowcytometry according to the indicated strategy for the presence of resident and migratory DCs.

Karthaus et al., Supplementary Figure 3

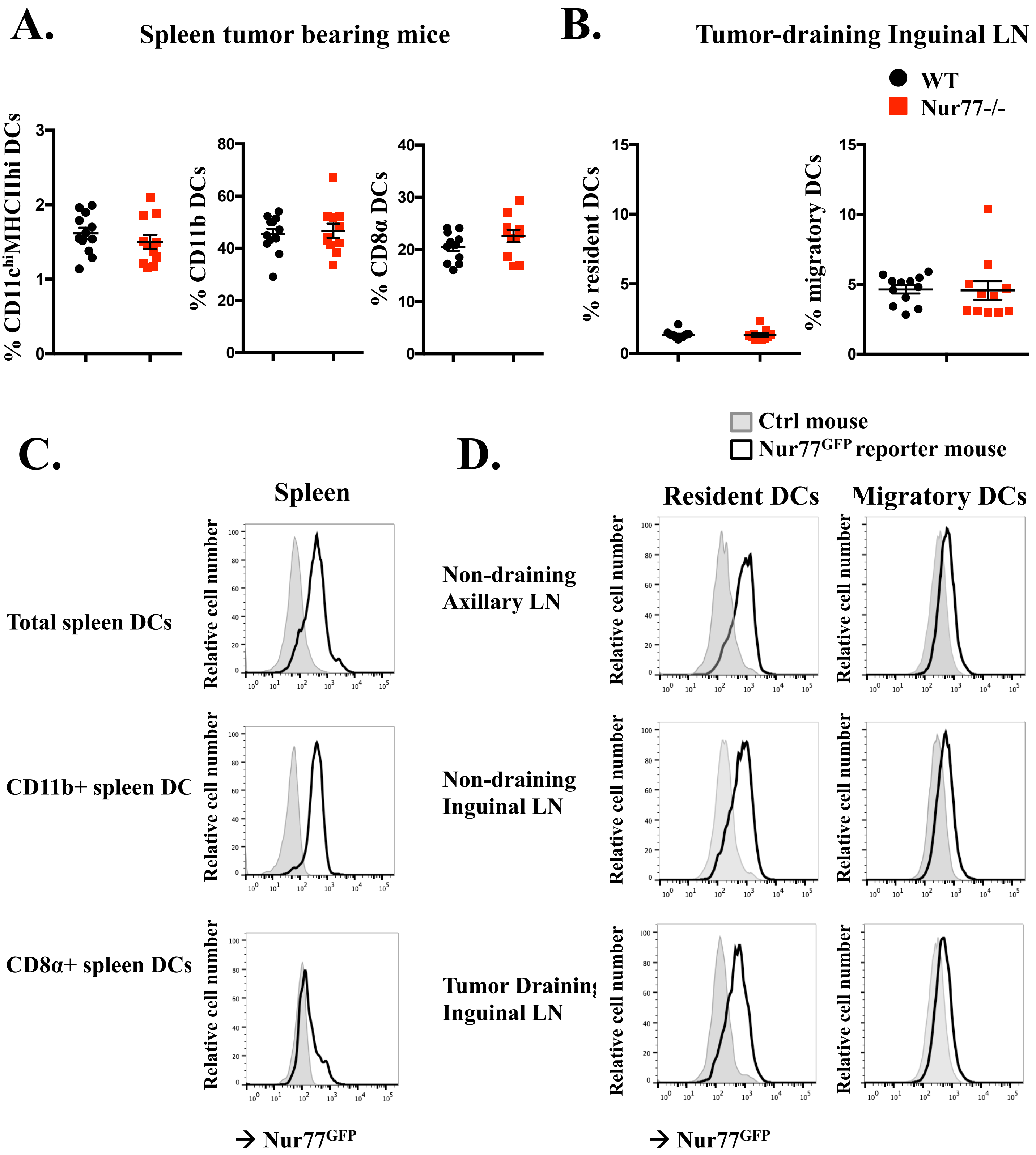

Supplementary figure 3. % of DCs in **A)** spleen and **B)** Tumor draining lymph nodes of WT or Nur77-/- mice bearing a 9464D neuroblastoma tumor. Shown are the pooled data of 2 independent experiments +/- SEM for WT or Nur77-/- mice (n = 4-6 WT or Nur77-/- mice per experiment). 2-tailed unpaired t test
Ex vivo Nur77^{GFP} expression in the different DC subsets in the **C)** spleen or **D)** non-draining axillary and inguinal and the tumor draining lymph nodes of 9464D tumor bearing mice. Shown are representative data of n = 3 mice/experiment from 2 independent experiments

Karthaus et al., Supplementary Figure 4

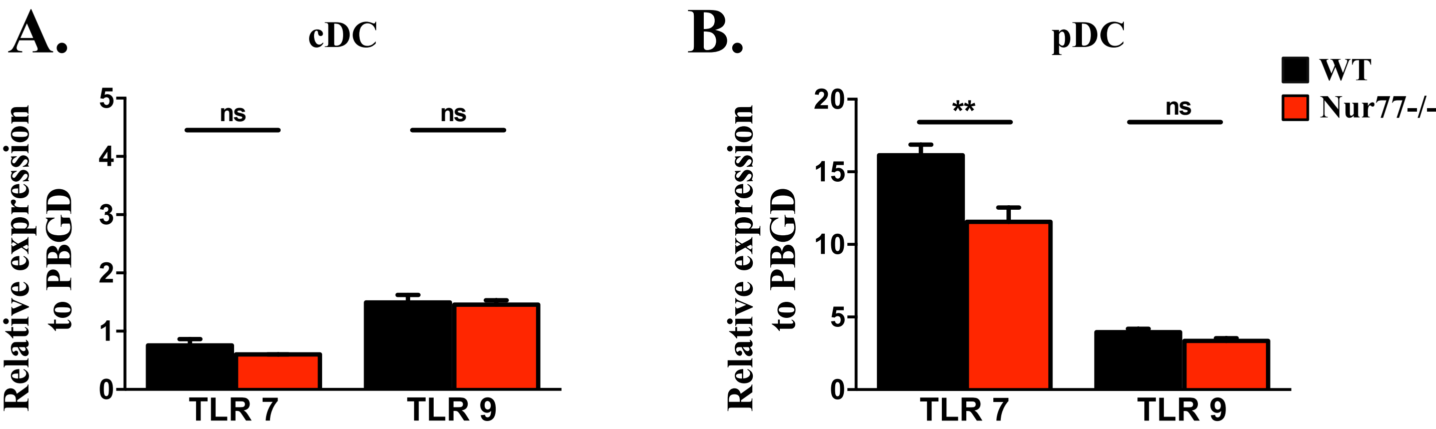

Supplementary Figure 4. TLR expression levels of murine Nur77-/- DCs. cDCs (A) and pDCs (B) were sorted from FLT3L BM cultures and immediately lysed for RNA isolation. mRNA expression levels of TLR7 and TLR9 were detected by qPCR analysis. Data shown are the mean of 3 mice +/- SEM, 2-way anova with bonferroni post test **P<0.01

Karthaus et al., Supplementary Figure 5

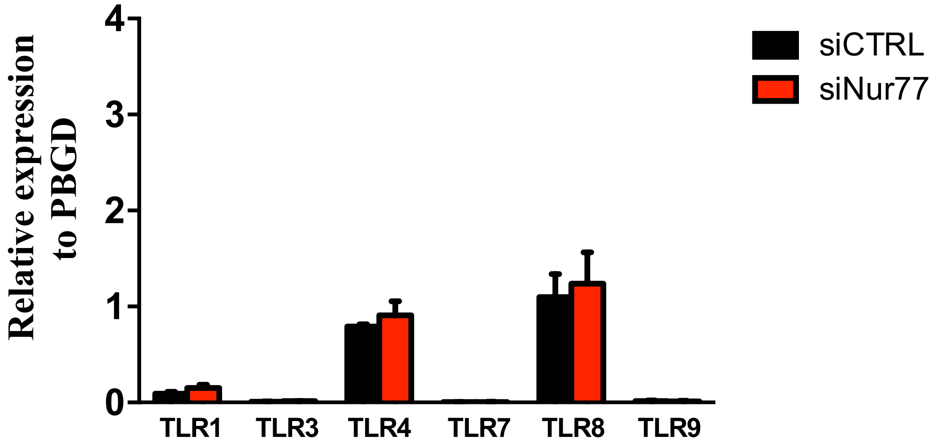

Supplementary Figure 5. TLR expression levels in siCTRL or siNur77 treated DCs. At day 6 of DC differentiation, DCs were lysed for RNA isolation. mRNA expression levels of TLR1, 3, 4, 7, 8 and 9 were detected by qPCR analysis. Data shown are the mean of 3 human donors +/- SEM

Karthaus et al., Supplementary Figure 6

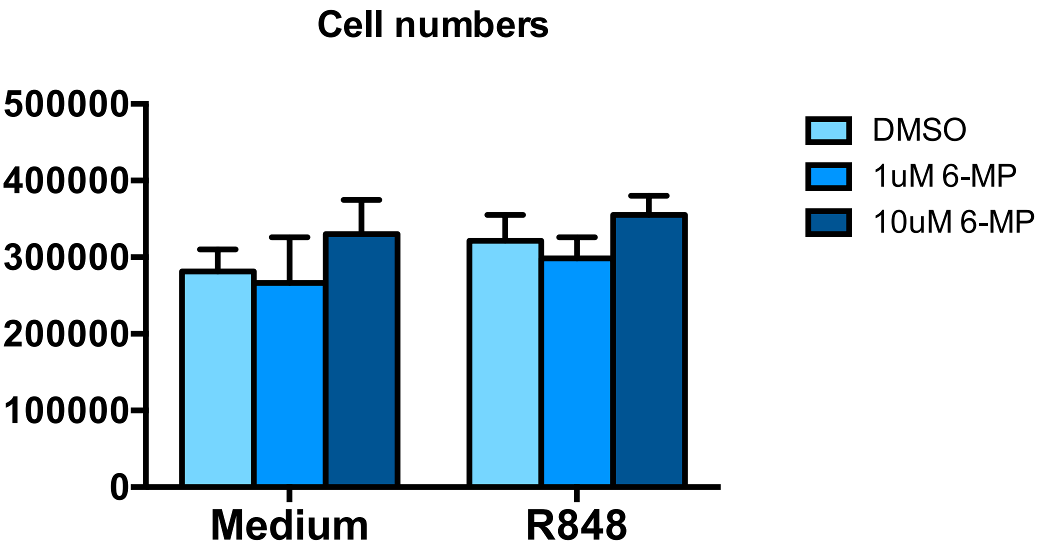

Supplementary figure 6. Treatment with 6-MP does not affect DC viability. moDCs were pretreated with 6-MP and stimulated with TLRL. After stimulation DC numbers were counted. Data shown are the mean of 3 human donors +/- SEM. 2-way anova with bonferroni post test
